# SCXRD, CSP-NMRX and microED in the quest for three elusive polymorphs of meloxicam

**DOI:** 10.1107/S2052252524011898

**Published:** 2025-01-01

**Authors:** Agata Jeziorna, Maura Malinska, Isaac Sugden, Piotr Paluch, Rafał Dolot, Marta K. Dudek

**Affiliations:** a Centre of Molecular and Macromolecular Studies Polish Academy of Sciences Sienkiewicza 112 Lodz90-363 Poland; b Faculty of Chemistry University of Warsaw Pasteura 1 Warsaw Poland; c Department of Chemical Engineering Imperial College London LondonSW7 2AZ United Kingdom; King Abdullah University, Saudi Arabia

**Keywords:** meloxicam, polymorphism, single-crystal X-ray diffraction, microcrystal electron diffraction, solid-state structures, crystal structure prediction, NMR crystallography

## Abstract

Crystal structure determination is a crucial aspect of almost every branch of the chemical sciences, bringing us closer to understanding crystallization, polymorphism, phase transitions, and the relationship between a structure and its physicochemical and functional properties. Unfortunately, many molecules notoriously crystallize as microcrystalline powders, providing a significant challenge in establishing their structures. In this work, we describe the crystal structure determination of three elusive polymorphs of the anti-inflammatory drug meloxicam (MLX) using three approaches, of which only one was successful for each crystal phase. Single-crystal X-ray diffraction allowed us to solve the structure of MLX-III, MLX-II was solved by a combination of NMR crystallography and crystal structure prediction (CSP) calculations, and MLX-V (*Z*′ = 4 polymorph) was only solvable using electron diffraction. By considering the factors influencing the choice of crystal structure determination method, we showcase their strengths and weaknesses as an indication of their applicability. Additionally, we discuss the issues encountered in the CSP search for MLX-II and MLX-III (both *Z*′ = 2 polymorphs) which turned out to be computationally elusive, in addition to being so in crystallization experiments. This indicates a complex crystal energy landscape for MLX and hints at more general challenges in CSP.

## Abstract 2

The case of three elusive polymorphs of meloxicam highlights the strengths and weaknesses of single-crystal X-ray diffraction, crystal structure prediction–NMR crystallography and microcrystal electron diffraction as crystal structure determination approaches. Each method was successful in solving only one of the polymorphs, showcasing the advantage of using the whole arsenal of available techniques.

## 1. Introduction

Crystal structure determination of drug polymorphs is one of the major challenges for the chemical sciences. In an ideal case, a single crystal of sufficient size can be grown to enable structure solution using single-crystal X-ray diffraction (SCXRD), which is still a method of choice in structural studies of solid forms. However, more often than not, the molecule of interest crystallizes in microcrystalline powder form, posing significant challenges in its crystal structure determination (Hušák *et al.*, 2019 ▸; Newman *et al.*, 2022 ▸). Then, alternative structure elucidation methods can be used, including solving the structure from powder X-ray diffraction (PXRD; Al Rahal *et al.*, 2021 ▸) microcrystal electron diffraction (microED; Gruene *et al.*, 2018 ▸) and NMR crystallography protocols, often together with crystal structure prediction (CSP-NMRX; Baias *et al.*, 2013 ▸; Dudek *et al.*, 2020*a* ▸). It is also possible to use a combination of these techniques in particularly difficult cases. For example, the crystal structure of a new polymorph of l-tyrosine has been recently solved using a combination of PXRD and ED measurements (Smalley *et al.*, 2022 ▸), while the CSP-NMRX approach together with PXRD yielded a crystal structure solution of form B of mebendazole (Bravetti *et al.*, 2022 ▸) and two polymorphs of furazidine (Dudek *et al.*, 2020*b* ▸). In this latter case, the established structures were later confirmed by SCXRD (Trimdale-Deksne *et al.*, 2023 ▸), adding to the credibility of the NMR-CSPX methodology. However, each of the crystal structure determination methods has its own limitations and none can be universally applied to solve all crystal structure determination issues.

For SCXRD to be used, well diffracting crystals of appropriate size and quality are required and this cannot always be achieved. The size of the crystal depends mostly on the kinetics of crystallization, of which we have an influence only to a certain extent. On top of that, not all crystallization techniques offer a chance for the crystals to grow. For example, mechanochemistry, vapour diffusion or desolvation are all well established crystallization techniques, often the only ones yielding elusive polymorphic forms (Bhardwaj *et al.*, 2019 ▸; Loya *et al.*, 2022 ▸), and all possibly leading to microcrystalline powders. Even having well diffracting crystals does not always result in a structure solution from SCXRD, *e.g.* because of twinning, modulation, diffuse scattering or instability of the crystal.

On the other side of the ‘crystal size’ palette is the microED approach, which is appropriate for nanocrystals, preferably thin ones, with an crystal thickness upper limit of 1 µm (Shi *et al.*, 2013 ▸; Martynowycz *et al.*, 2019 ▸). This limit can be attributed to the large dynamic scattering effect, resulting in a small penetration depth of the crystal by the electron beam. On top of that, microED measurements are associated with significant radiation damage on the sample, thus forcing the measurements to be quick and leading to a necessity of merging the data obtained from at least several crystals (Das *et al.*, 2018 ▸; Huang *et al.*, 2021 ▸).

Also partly associated with crystallite sizes, but perhaps more with their shape, is the PXRD preferred orientation problem, able to severely disrupt the intensity of reflections, leading to difficulties in Rietveld refinement (Smalley *et al.*, 2022 ▸). For this technique, there is also an issue of ambiguity of structure solution, such as the one recently encountered for 4,11-di­fluoro­quinacridone, for which PXRD data showed a good fit to four different structural models (Schlesinger *et al.*, 2022 ▸).

Finally, the CSP-NMRX protocols suffer from several issues, including ambiguities in signal assignment and/or extracting informative-enough constraints from solid-state NMR spectra, as well as access to the know-how and state-of-the-art spectrometers allowing for measurements with very high spinning speed sample rotation. Combining solid-state NMR experiments with CSP calculations can alleviate some of these issues (Bravetti *et al.*, 2022 ▸; Dudek *et al.*, 2020*a* ▸; Dudek *et al.*, 2020*b* ▸), but for flexible, multi-component or high-*Z*′ systems, there is often a prohibitively large search space to cover in a reasonable time (Bowskill *et al.*, 2021 ▸). Being aware of all these limitations but at the same time also of the advantages of using each crystal structure determination technique can lead to rational selection of the best tool to tackle a particular structural issue.

In this work, we demonstrate a unique case of three neat polymorphs of meloxicam (MLX) we were able to crystallize (Jeziorna *et al.*, 2023 ▸) that to date have eluded all crystal structure determination attempts, despite being reported for the first time 20 years ago (Coppi *et al.*, 2003 ▸). Commercially, MLX is sold in its most thermodynamically stable form, MLX-I, for which the crystal structure was established in 1998 (Fabiola *et al.*, 1998 ▸). The remaining elusive forms, MLX-II, MLX-III and MLX-V, proved to be very challenging in crystal structure determination, and each required a different characterization technique: SCXRD (MLX-III), a combination of CSP-NMRX with PXRD (MLX-II), and microED (MLX-V). Note that for each form only one crystal structure determination method was fully successful.

## 2. Results and discussion

### 2.1. Meloxicam structure

**Figure 1:**
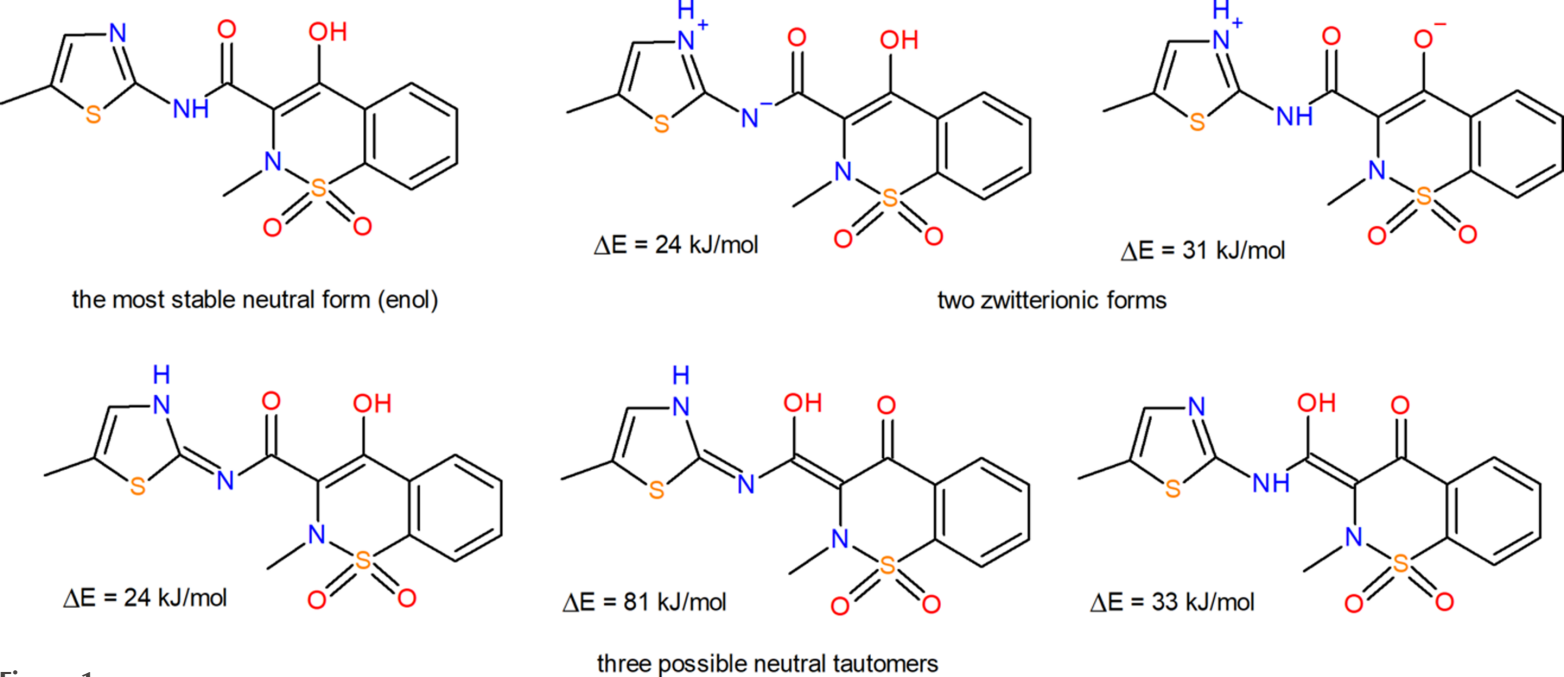
Chemical structure of MLX in its most thermodynamically stable enolic form, as well as five possible tautomeric forms, two of which are zwitterionic. Next to each possible tautomeric form is its relative gas-phase energy with respect to the most stable form. The energies were calculated at the B3LYP-GD3BJ/6-311G(d,p) level of theory.

MLX (Fig. 1 ▸) is prone to tautomerism, resulting in several possible molecular forms. In the gas phase the most stable is its neutral enol form, and the other possible forms are significantly less stable, which means it is unlikely they can be found in any MLX crystals. This, however, does not concern zwitterionic forms which, despite being less energy stable in the gas phase, can be accommodated in a crystal structure because ionic interactions are better able to compensate for the unfavourable conformational energy than non-ionic ones. Our recent survey for zwitterionic and non-zwitterionic crystals of piroxicam, a molecule closely related to MLX, has shown that crystal structures can be built by zwitterionic conformations with a gas-phase energy of up to at least 42 kJ mol^−1^ higher than the gas-phase minimum conformation and that lattice energies of such zwitterionic-built structures (understood as the contribution of the intermolecular interactions to the total energy) are on average 13% lower than the neutral ones (Jeziorna *et al.*, 2023 ▸). For MLX this means that low-energy crystal structures can be built by both neutral and zwitterionic forms.

### 2.2. Preliminary characterization of polymorphs

The identities of MLX-II, MLX-III and MLX-V were confirmed by comparison of their PXRD patterns with those disclosed in the patent literature (Coppi *et al.*, 2003 ▸) (Fig. S1 of the supporting information). We have already reported the ^13^C and ^15^N CPMAS NMR spectra for these forms (Jeziorna *et al.*, 2023 ▸), suggesting that all three contain at least two symmetry-independent molecules in the asymmetric part of the crystallographic unit cell (*Z*′ > 1 polymorphs), which is visible as a doubling of some of the resonances. Additional 2D NMR ^1^H–^13^C HETCOR spectra registered under very fast MAS conditions suggested that MLX-V can be a *Z*′ = 4 polymorph, as some of the correlation signals appeared to correspond to four different sites (see Fig. S2). Thermogravimetric analyses for these forms confirmed that all are neat polymorphs (Fig. S3), whereas DSC measurements (Fig. S4) showed that MLX-I and MLX-III are monotropically related with MLX-III transforming to MLX-I at around 200°C, as already suggested on the basis of DSC analysis of a mixture of concomitantly crystallized MLX-I and MLX-III (Freitas *et al.*, 2017 ▸). Similarly, MLX-II and MLX-V are monotropically related, with the phase transition taking place already at *ca* 139°C. These results are in line with our earlier observations of MLX-II easily transforming to MLX-V during desolvation at increased temperatures (Jeziorna *et al.*, 2023 ▸).

### 2.3. Selecting the crystal structure determination method

In our studies, MLX-II and MLX-V always crystallized as very fine microcrystalline powders, precluding the use of SCXRD. Despite many efforts, most of the PXRD diffractograms of MLX-II contained some small admixtures of MLX-V, posing a challenge for structure solution from powder. Even if MLX-V was not primarily present in the analysed sample, we saw signs of the transformation from MLX-II to MLX-V after roughly 30 min (this, however, depended on whether the sample was obtained by the dehydration of a hydrate or desolvation of an HFIP solvate, as well as on the humidity and temperature conditions currently present in the laboratory), hampering the indexing results. Fortunately, the registered solid-state NMR spectra were of sufficient quality to attempt NMR-CSPX crystal structure determination (the presence of resonances originating from MLX-V was visible in the registered spectra, but they were of lower intensity and did not hinder the assignment of signals to MLX-II), in particular, the CSP calculations conducted by us previously (Jeziorna *et al.*, 2023 ▸) resulted in finding three structural models with simulated PXRD patterns resembling the experimental one for MLX-II (see below). To indicate the correct model we used the NMRX approach, described in the following section.

In the case of MLX-V, the registered NMR spectra indicated a resemblance of this structure to that of MLX-II, but at the same time they also hinted at a higher *Z*′ value of this polymorph (possibly *Z*′ = 4). Tackling such a structure with CSP would be prohibitively time- and resource-consuming. We were also unable to reliably index the PXRD diffractogram of MLX-V and so we marked MLX-V as the best candidate for microED crystal structure determination.

Finally, in the case of MLX-III, in our earlier experiments we noticed that this form is by far the most frequently occurring one during crystallization from aqueous solutions of sodium hydroxide on the addition of 80% acetic acid. In the majority of such crystallization attempts, MLX-I was primarily formed, but many times concomitantly with other polymorphs (Jeziorna *et al.*, 2023 ▸). On top of that, this procedure in a slightly modified form was reported by the patent literature to lead to elusive forms of MLX, including MLX-III (Coppi *et al.*, 2003 ▸). Since such manner of crystallization offers a better chance to facilitate crystal growth, we tried to use it to obtain SCXRD-suitable crystals of one of the elusive MLX forms. Indeed, in one of the attempts, in which the supersaturated aqueous solution of MLX was left in a chamber with toluene vapour, small crystals were formed, later identified as a mixture of MLX-I and MLX-III. This enabled us to use SCXRD in the crystal structure determination of this polymorph.

### 2.4. CSP-NMRX of MLX-II

**Figure 2:**
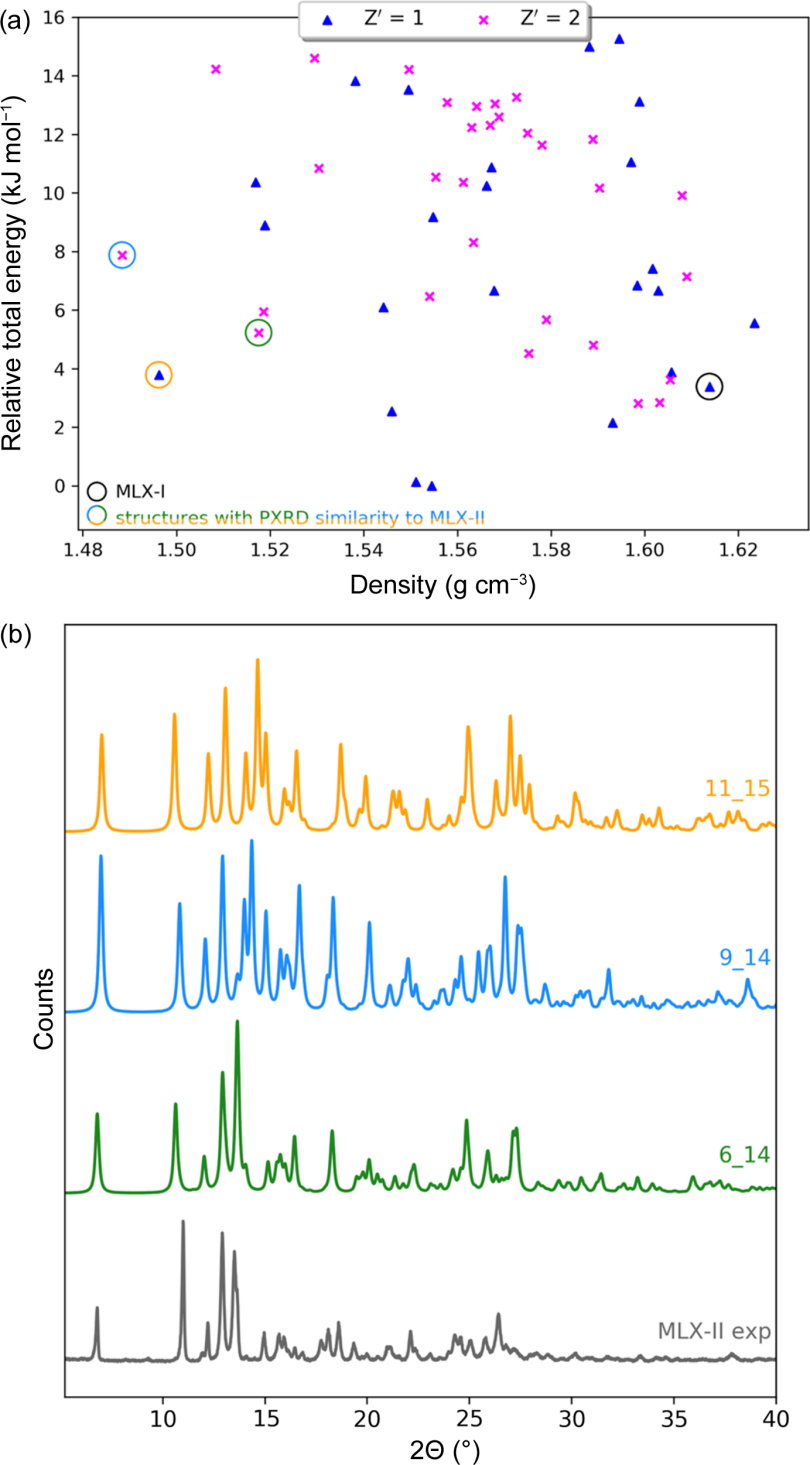
(*a*) Lowest-energy part of the PBE-MBD* crystal energy landscape of MLX with possible structure candidates for MLX-II marked with orange/blue/green circles; the black circle marks the most thermodynamically stable MLX-I (CCDC refcode SEDZOQ). (*b*) Comparison of the experimental PXRD pattern of MLX-II with the simulated ones of three structural candidates obtained from CSP calculations [PXRD pattern colours correspond to those of the circles marking the structures in (*a*)].

The lowest part of the CSP–DFT–MBD energy landscape (up to 15 kJ mol^−1^ above the global minimum structure) obtained for *Z*′ = 1 and *Z*′ = 2 searches in the 40 and 10 most common crystallographic space groups (SGs) (Jeziorna *et al.*, 2023 ▸), respectively, contains 55 distinct crystal structures, of which 30 are *Z*′ = 2 (Fig. 2 ▸). The first step to identify the most likely candidates of a given polymorph is a comparison of the simulated PXRD patterns with the experimental one (in this case MLX-II). On the basis of this comparison, we selected three structures for which the respective diffractograms are shown in Fig. 2 ▸(*b*). Two of those structures have *Z*′ = 2, designated *6_14* and *9_14* (the naming scheme for the structures follows original rank of the structure from the force-field CSP plot in a given SG so that the first number is the structure rank within a given SG and the second one is the SG number; structure *6_14* is therefore the sixth structure according to its force-field relative energy in the SG *P*2_1_/*c*), whereas the third has *Z*′ = 1 and is named *11_15*. Therefore, the first thing to be established is the *Z*′ number for the experimental MLX-II and this can often be done using solid-state NMR spectra (Dudek *et al.*, 2018 ▸), although caution is advised as sometimes NMR-timescale dynamics may lead to incorrect conclusions (Widdifield *et al.*, 2017 ▸).

**Figure 3:**
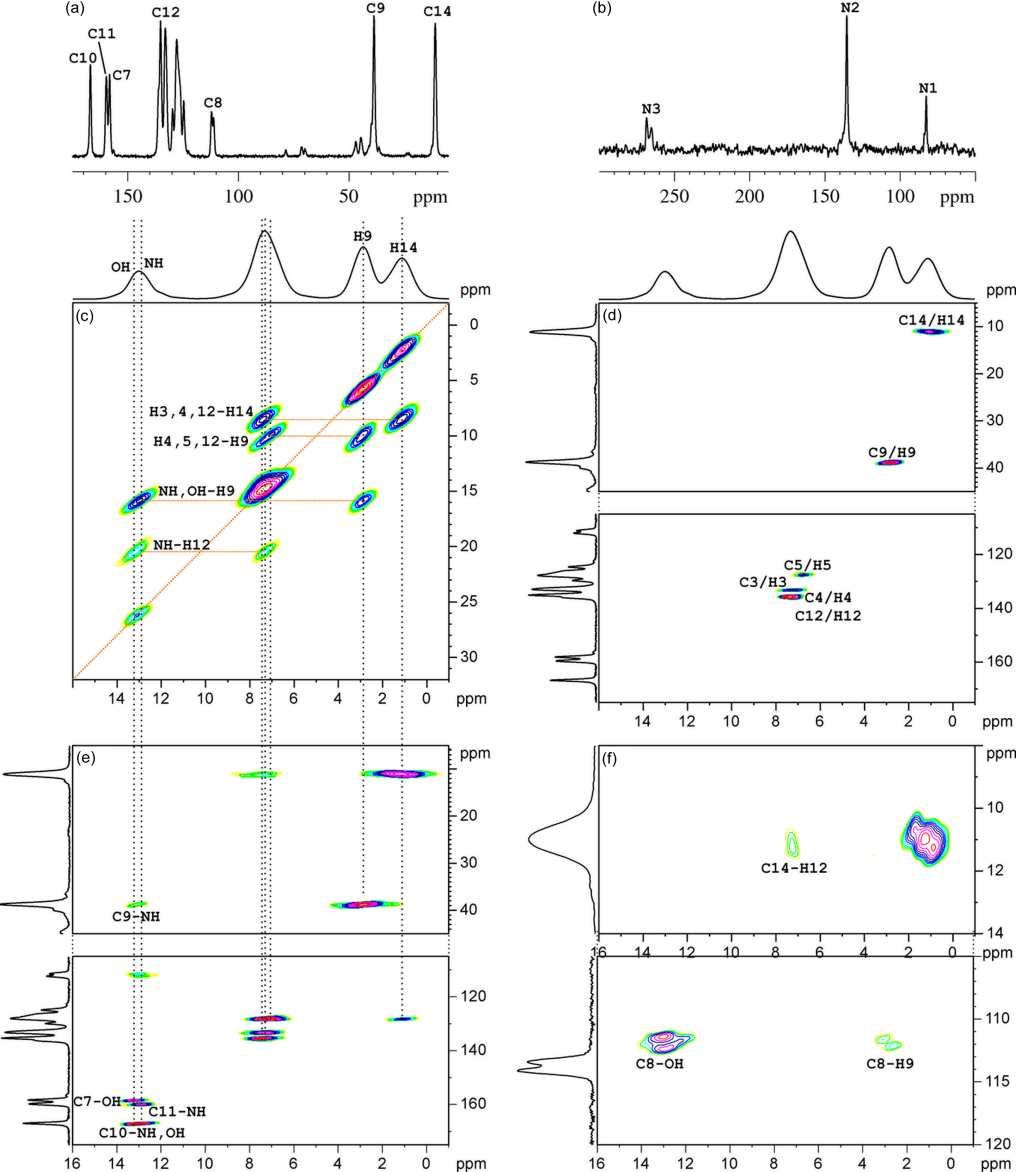
Solid-state NMR spectra for MLX-II. (*a*) ^13^C and (*b*) ^15^N CPMAS registered with ν_r_ = 13.33 kHz, (*c*)–(*f*) correlation spectra registered under very fast magic angle spinning conditions (ν_r_ = 55.55 kHz). (*c*) ^1^H–^1^H SQ–DQ correlation with back-to-back (BaBa) recoupling. (*d*)–(*f*) inv-^1^H–^13^C HETCOR with (*d*) short and (*e*)–(*f*) long contact time. The F1 projection in (*d*)–(*f*) shows the ^13^C CPMAS NMR spectrum registered with ν_r_ = 13.33 kHz, whereas F2 in (*c*)–(*d*) is the ^1^H MAS spectrum registered with ν_r_ = 55.55 kHz. The signal assignment of the most important resonances is shown in the respective spectra, and the full assignment is given in Table S1.

Figs. 3 ▸(*a*)–3 ▸(*b*) feature ^13^C and ^15^N CPMAS NMR spectra of MLX-II. Although the majority of the ^13^C resonances are not split, there is one signal at *ca* 112 p.p.m. arising from the C8 carbon. Admittedly, this signal is directly attached to the N1 nitro­gen atom and so the doubling of the C8 resonance could arise from the dipolar interaction with the ^14^N quadrupole moment of N1 (Hexem *et al.*, 1981 ▸). However, there is also a clearly visible doubling of one of the ^15^N resonances, originating from the N3 nitro­gen, for which no similar explanation can be given. Additionally, a small splitting of the ^1^H–^13^C correlation peaks for C8, methyl and Ar—H resonances [Fig. 3 ▸(*f*)] is visible. All this adds up to a conclusion that *Z*′ = 2 for the MLX-II crystal and, as a result, the *11_15* model can be excluded from further considerations.

A close resemblance of all chemical shifts arising from two distinct molecules in an asymmetric part of the unit cell of form II suggests that they both assume a very similar or indeed the same conformation and share a similar chemical surrounding. In contrast, some of the chemical shifts observed for MLX-I (see Table S1 of the supporting information) are markedly different. In particular, the ^1^H chemical shift of the NH hydrogen is equal to 13.2 p.p.m. for both molecules of MLX-II and 9.2 p.p.m. for MLX-I, which clearly indicates a different hydrogen bonding pattern, as already suggested by the analysis of 1D spectra (Jeziorna *et al.*, 2023 ▸). On the other hand, in both polymorphs the ^1^H chemical shift value for the OH proton is practically the same and equal to 12.7–12.9 p.p.m., pointing to a similar OH surrounding in both structures. The analysis of two *Z*′ = 2 structural models from CSP shows that in both cases the structures are stabilized by NH⋯N hydrogen bonds, while in MLX-I NH⋯O=S hydrogen bonds are observed.

**Figure 4:**
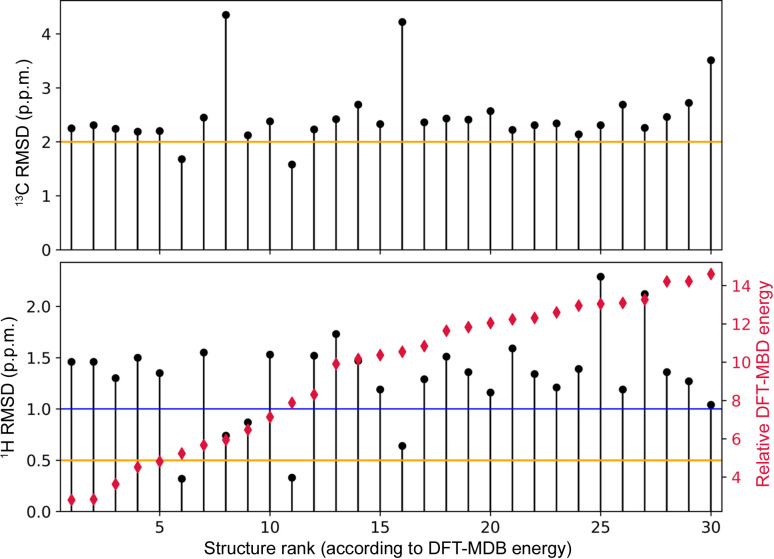
^1^H and ^13^C RMSD values from a comparison of the experimental chemical shifts of MLX-II with those calculated for all *Z*′ = 2 low-energy structures from the CSP energy landscape, together with their total DFT–MBD energies (in kJ mol^−1^) relative to the global minimum structure. The orange lines show the RMSD cut-off values of 0.5 and 2 p.p.m. for ^1^H and ^13^C, respectively; the blue line indicates the cut-off of 1 p.p.m. for ^1^H NMR data. For numerical values used to plot the data, see Table S2.

**Figure 5:**
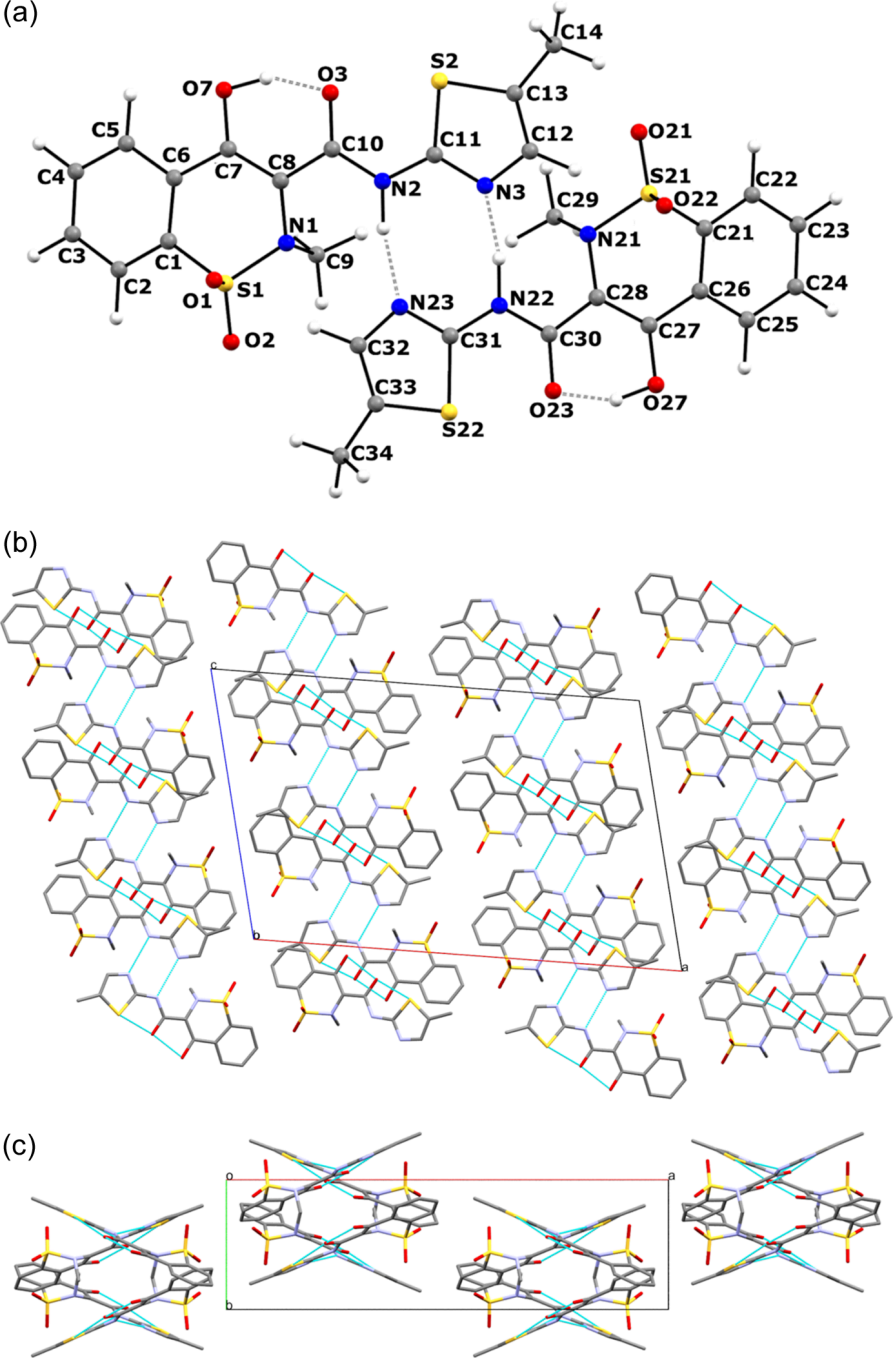
(*a*) NH⋯H dimer unit building the CSP-predicted crystal structure of MLX-II together with atom numbering scheme. Crystal packing diagram of MLX-II along the (*b*) *b* and (*c*) *c* axes.

For both candidate structures, as well as for all other low-energy *Z*′ = 2 CSP models, ^1^H and ^13^C chemical shifts were calculated and compared with the experimental ones. Fig. 4 ▸ shows a comparison of the ^1^H and ^13^C chemical shift RMSD values (in p.p.m.) obtained for these structures, featuring differences in the level of agreement between experimental and theoretical values. The expected level of this agreement for a viable model can be slightly different for different systems (Bravetti *et al.*, 2022 ▸; Dudek *et al.*, 2020*b* ▸; Hofstetter *et al.*, 2019 ▸), but typically RMSD values below the 0.5 and 2 p.p.m. thresholds for ^1^H and ^13^C, respectively, are expected (Hofstetter & Emsley, 2017 ▸; Widdifield *et al.*, 2020 ▸). The only two structures falling below these two cut-off values are the *6_14* (6th structure in the plot) and *9_14* (11th structure in the plot) models, with ^1^H RMSD values of 0.32 and 0.33 p.p.m., respectively. These are the same structure candidates that were indicated by PXRD data analysis earlier. The comparison of these two structures using the *Crystal Packing Similarity Tool* (Chisholm & Motherwell, 2005 ▸) quite expectedly shows that they share 12 molecules out of the 15-molecule cluster with an RMSD_12_ value for atomic positions of 0.364 Å. Such a close structural match of the candidate structures often results in similar RMSD values for NMR data (Bravetti *et al.*, 2022 ▸; Engel *et al.*, 2019 ▸). Which structure is then closer to the experimental one? The first criterion is the energy difference between the candidates, with *6_14* being 2.65 kJ mol^−1^ of molecules lower in energy than *9_14*. On top of that, *6_14* shows a closer match to the experimental PXRD data and its two symmetry-inequivalent molecules assume almost identical conformation, in agreement with NMR data, while in *9_14* the conformation of one of the molecules is slightly distorted, although the attempted Rietveld refinement was not conclusive in this case. Finally, we would like to point out that at finite temperatures both structures may actually converge to the same minimum (Francia *et al.*, 2021 ▸; Yang & Day, 2022 ▸), and their presence at the 0 K CSP landscape can be an example of CSP overprediction (Francia *et al.*, 2020 ▸; Butler & Day, 2023 ▸). Therefore, the *6_14* candidate structure is designated as corresponding to the experimental structure of MLX-II. It has an SG symmetry of *P*2_1_/*c* and, as expected, the two MLX molecules in this structure form NH⋯N dimers [Fig. 5 ▸(*a*)], in which two interacting entities belong to different crystallographic planes [Figs. 5 ▸(*b*) and 5 ▸(*c*)]. As a result, the MLX-II dimers are less efficient in packing than planar NH⋯O=S dimers present in MLX-I, leading in consequence to less dense crystal structures (Jeziorna *et al.*, 2023 ▸). The two molecular conformations of MLX inside this crystal are almost identical and differ only slightly from the conformation found in MLX-I, with the RMSD of the atomic positions equal to 0.05 Å (Fig. S5).

In CSP-NMRX applications, it is always worth looking at all structure candidates that yield reasonable agreement with the NMR experiment to gain an understanding of the influence that differences in structural features have on NMR chemical shifts. In Fig. 4 ▸, a blue line marks a 1 p.p.m. cut-off for RMSD values in terms of ^1^H chemical shifts and there are five structural models falling below this cut-off: two models considered above and candidates with rank 8th (model *31_2*), 9th (model *16_14*) and 16th (*36_2* model). Two of those (8th and 16th), having ^1^H RMSDs of 0.74 and 0.64 p.p.m., respectively, are built by one neutral and one zwitterionic molecule, and so their hydrogen bond pattern is quite different from MLX-II. Still, NH and OH groups participate in the strong hydrogen bonds, just like in MLX-II. On the other hand, the ^13^C RMSD values for these two structures are well above any acceptable values (>4.0 p.p.m.). In this case it seems that the agreement in terms of ^13^C is more discriminative than usual. The remaining 9th structure (*16_14*) shares the NH⋯N hydrogen bond motif and 6 out of 15 molecules (atomic RMSD_6_ of 0.46 Å) with MLX-II when compared using the *Crystal Packing Similarity Tool*. Note, none of the other structure candidates are stabilized by this hydrogen bond motif.

### 2.5. SCXRD success and CSP-NMRX failure in MLX-III crystal structure determination

Similar to MLX-II, solid-state NMR spectra for MLX-III indicated it is a *Z*′ = 2 polymorph, possibly sharing the same hydrogen bonding pattern as MLX-II and possibly also the molecular conformation (Fig. S6). Therefore, having in hand quite a thorough CSP search, we searched the pool of candidate structures for those with a PXRD pattern similar to that of MLX-III, but did not find any viable candidates. In these circumstances, one can either go back to computations to try to search for unaccounted degrees of freedom, or try experimental methods of crystal structure determination. We did both, but primarily succeeded only in the latter. Only in the single-crystal structure determination of MLX-III were we able to identify the reason for failure of the first CSP calculations and find a remedy.

In the vast majority of cases, MLX crystallizes in the form of a very fine powder, but some of the patent-inspired crystallization attempts resulted in a concomitant crystallization of MLX-I together with small crystals of somewhat different morphology: those corresponding to MLX-I were larger rhomboid prisms, while the others were smaller, also resembling prisms, but were more elongated and had a pointed end. This enabled us to use SCXRD for their structural determination. Possible reasons for our failure in finding the correct structural model of MLX-III from the blind CSP are discussed below the description of the SCXRD results, in light of the knowledge of what the structure looks like.

**Figure 6:**
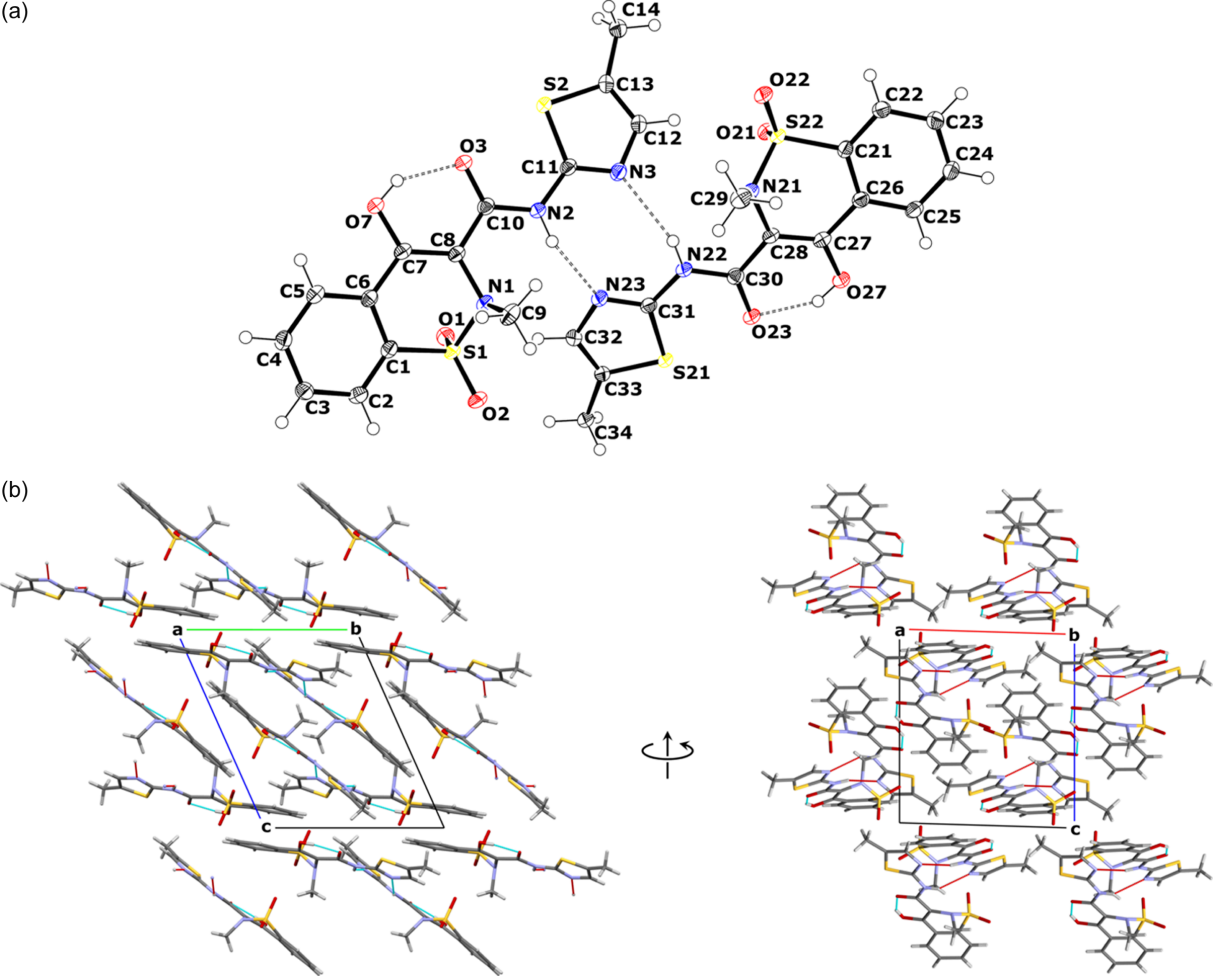
(*a*) Asymmetric unit of the MLX-III crystal showing the atom-labelling scheme and inter- and intramolecular hydrogen bonds. Displacement ellipsoids are drawn at the 50% probability level. (*b*) Crystal packing diagram of MLX-III. The view along the *a* axis is visible on the left and the view along the *b* axis is on the right.

The crystallographic details for MLX-III are shown in Table 1 ▸, and all additional data are gathered in the supporting information (Tables S3–S9). The third form of MLX was found to crystallize in the triclinic SG *P*1 with its asymmetric unit consisting of two molecules of MLX in its neutral form [Fig. 6 ▸(*a*)]. Both molecules are interlinked by two weak hydrogen bonds, N2—H2⋯N23 and N22—H22⋯N3, and form a dimer of the *R*_2_^2^(8) motif, as expected from the solid-state NMR results. In both MLX molecules there is an intramolecular hydrogen bond present between O7—H7⋯O3 and O27—H27⋯O23, forming *S*(6) motifs, as observed in MLX-I and MLX-II. There are no additional hydrogen bonds with symmetry-related molecules and the MLX dimers are arranged alternately in intersecting planes [Fig. 6 ▸(*b*)]. Importantly for further discussion, both symmetry-independent molecules assume a very similar conformation to each other, as well as to the conformation found in MLX-I and MLX-II. In fact, an overlay of molecules A and B with a molecule from MLX-I yields RMSD values for atomic positions of 0.107 and 0.115 Å, respectively. As will be shown later, this almost negligible difference turned out to be crucial for the CSP calculations.

Having revealed the structure of MLX-III, we can shortly discuss the issue of not finding a reasonable model of this structure in our primary CSP calculations. This first attempt was made using a rigid-body search (the molecular conformation of MLX was not allowed to vary at the crystal structure generation stage) and allowing molecular flexibility at the geometry optimization stage. This is one of the commonly used approaches in CSP (Dudek & Drużbicki, 2022 ▸), in particular for cases like MLX, when spectroscopic evidence suggests a similarity of molecular conformation in all analysed crystal phases. Analysing this rigid-body CSP, first it should be noted that the search was performed for a set of SGs including the correct one. Also, one of the molecular conformations of MLX taken for the calculations was very similar to that present in the crystal. This was the conformation of the most stable gas-phase conformer of MLX, which differs in terms of RMSD values for the atomic position by 0.177 and 0.188 Å from the two MLX-III molecules. Furthermore, both MLX-I and MLX-II were found in this CSP and their relative total energies were among the lowest values. In particular, in the force-field energy landscape, MLX-I was marked as the global minimum structure, while MLX-II was 5 kJ mol^−1^ higher in energy. This means that the force-field parameters used for the geometry optimization represent the evaluated systems well. Fourthly, the CSP convergence was ensured by examining how many times each crystal structure was found in the search and the calculations were continued until each low-energy structure was found at least twice. Extending the search for more crystal structures (from 10 000 to 100 000 valid crystal structures) did not help in finding MLX-III on the landscape. Finally, and most importantly, after taking two molecular conformations of MLX directly from the experimentally determined crystal for CSP calculations, we did easily find MLX-III when generating a standard number of 10 000 trial crystal structures, with a relative lattice energy of 4.68 kJ mol^−1^ above the global minimum structure. All this points to a molecular conformation being the culprit here. Usually, such differences in molecular conformation as those reported between MLX-III conformations and the gas-phase minimum conformation used in the primary CSP search are perfectly negligible and are later corrected at the stage of the DFT geometry optimization (Dudek & Drużbicki, 2022 ▸). Here, they seem to be decisive and may indicate the necessity of accounting for molecular flexibility at the crystal-generation stage. Indeed, flexible CSP with *CrystalPredictor* (Habgood *et al.*, 2015 ▸) was able to find two polymorphs (MLX-I and MLX-III), but missed MLX-II in a default workflow, demonstrating the challenging energetic landscape for this *Z*′ = 2 system. Clearly, for MLX even small distortions in molecular conformation introduced at the crystal structure generation stage resulted in finding a missing structure of MLX-III. Somewhat similar observations were made before for phenobarbital (Day *et al.*, 2007 ▸), whereby to find one of the polymorphic forms using CSP without flexibility included at the crystal structure generation stage, it was necessary to start calculations not with any of the gas-phase minimum conformations, but rather with a saddle-point conformation.

The reported conformational issues in CSP calculations have been observed before (Trimdale-Deksne *et al.*, 2023 ▸; Braun *et al.*, 2019 ▸; Cruz Cabeza *et al.*, 2006 ▸). For example, in the case of gandotinib, forms I and II of this molecule were not found in a CSP search. Instead, for form I many similar crystal structures were discovered in the CSP landscape and so the lack of an exact match to the experimental structure was attributed to a conformational disorder observed in this crystal, confirmed by solid-state NMR experiments (Braun *et al.*, 2019 ▸). Contrarily, the absence of form II in the CSP-generated set of structures was explained by its relatively high energy, resulting in this structure lying in the region of poorer CSP convergence. In the case of MLX-III, however, no disorder was observed in either the single-crystal X-ray diffraction or the solid-state NMR experiments. Also, the overly rigorous energy cut-off was determined not to be an issue here. In another report, in which a molecular conformation used in a CSP search was not an exact match to the experimental conformation, the experimental crystal structures were found, but they were lying much higher on the CSP energy landscape, as their energies were not well reproduced (Cruz Cabeza *et al.*, 2006 ▸). This is also not the case for MLX-III, as the search for the structural model of this polymorph among higher-energy structures (with relative energies of up to 50 kJ mol^−1^) also did not result in a match. Finally, we observed a very similar problem in finding the furazidin form III polymorph from CSP calculations, unless a very exact molecular conformation was used in the rigid CSP search (Trimdale-Deksne *et al.*, 2023 ▸). We hypothesize that these computationally elusive polymorphs lie in a very shallow energy minimum. Once a slightly different conformation is used in the rigid crystal structure search, the generated structures converge to a much deeper minimum of the most stable polymorphic form. Further calculations are planned to fully reveal the reasons behind the severity of molecular conformation influence on the CSP predictions for MLX and furazidin.

### 2.6. microED of MLX-V

**Figure 7:**
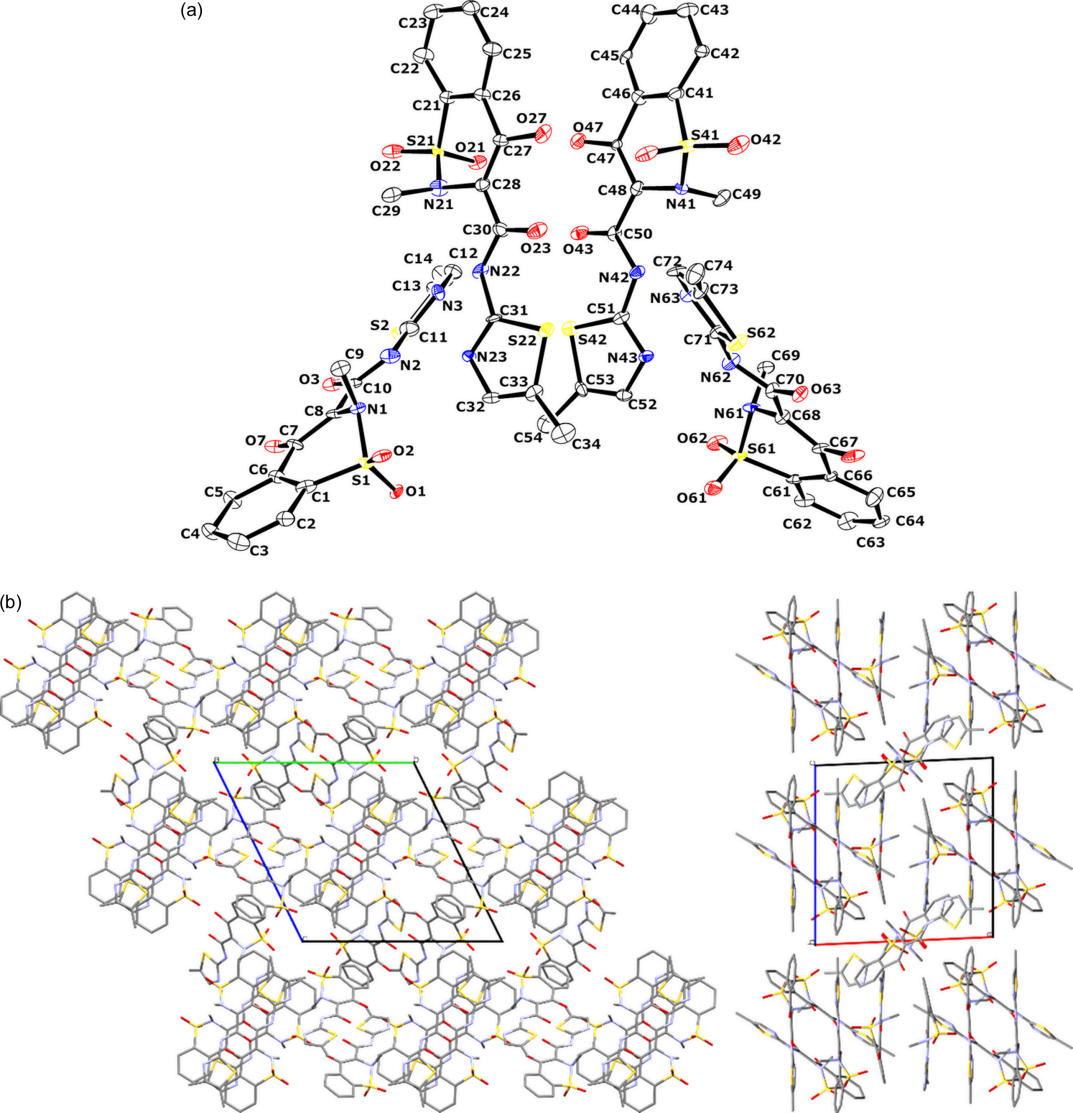
(*a*) Asymmetric unit of the MLX-V crystal showing the atom labelling. Atom displacement parameters are drawn at the 50% probability level. (*b*) Crystal packing diagram of MLX-V. The view along the *a* axis is visible on the left, and the view along the *c* axis is on the right.

MLX-V crystallizes as a fine powder with plate-like single crystals ranging in size between 100 and 200 nm. Therefore, only electron diffraction techniques can reveal the structure from a single particle. The experimental details for MLX-V are shown in Table 2 ▸, and all additional data are gathered in the supporting information (Tables S12–S18). The fifth form of MLX was found to crystallize in the triclinic SG *P*1, with its asymmetric unit consisting of four molecules of MLX in its neutral form, forming a pair of dimers [Fig. 7 ▸(*a*)]. In all MLX molecules, there is an intramolecular hydrogen bond, as observed in MLX-I, II and III. These dimers interact through two N—H⋯N hydrogen bonds, similar to MLX-II and MLX-III. The first dimer forms via N2—H2⋯N23 and N22—H22⋯N3, whereas the second one connects via N42—H42⋯N63 and N62—H62⋯N43. Both dimers are interlinked by stacking, forming intersecting planes with angles of 55.7 and 56.2° calculated based on non-hydrogen atoms for each molecule in the dimer for the first and second pairs. However, the stacking contacts are different compared with MLX-III. In MLX-V, stacking contacts involve a face-to-face orientation of thia­zole rings, whereas in MLX-III, the thia­zole ring interacts with the carbonyl bond of the second molecule. These arrangements result in the formation of small discrete voids with a volume of 8 Å^3^, surrounded by sulfonyl groups, making it hydro­philic [Fig. 7 ▸(*b*)]. It is also consistent with this crystal form being easily accessible via desolvation of the HFIP solvate or MLX hydrate (Jeziorna *et al.*, 2023 ▸).

### 2.7. Evaluation of energetic stability of MLX polymorphs

**Figure 8:**
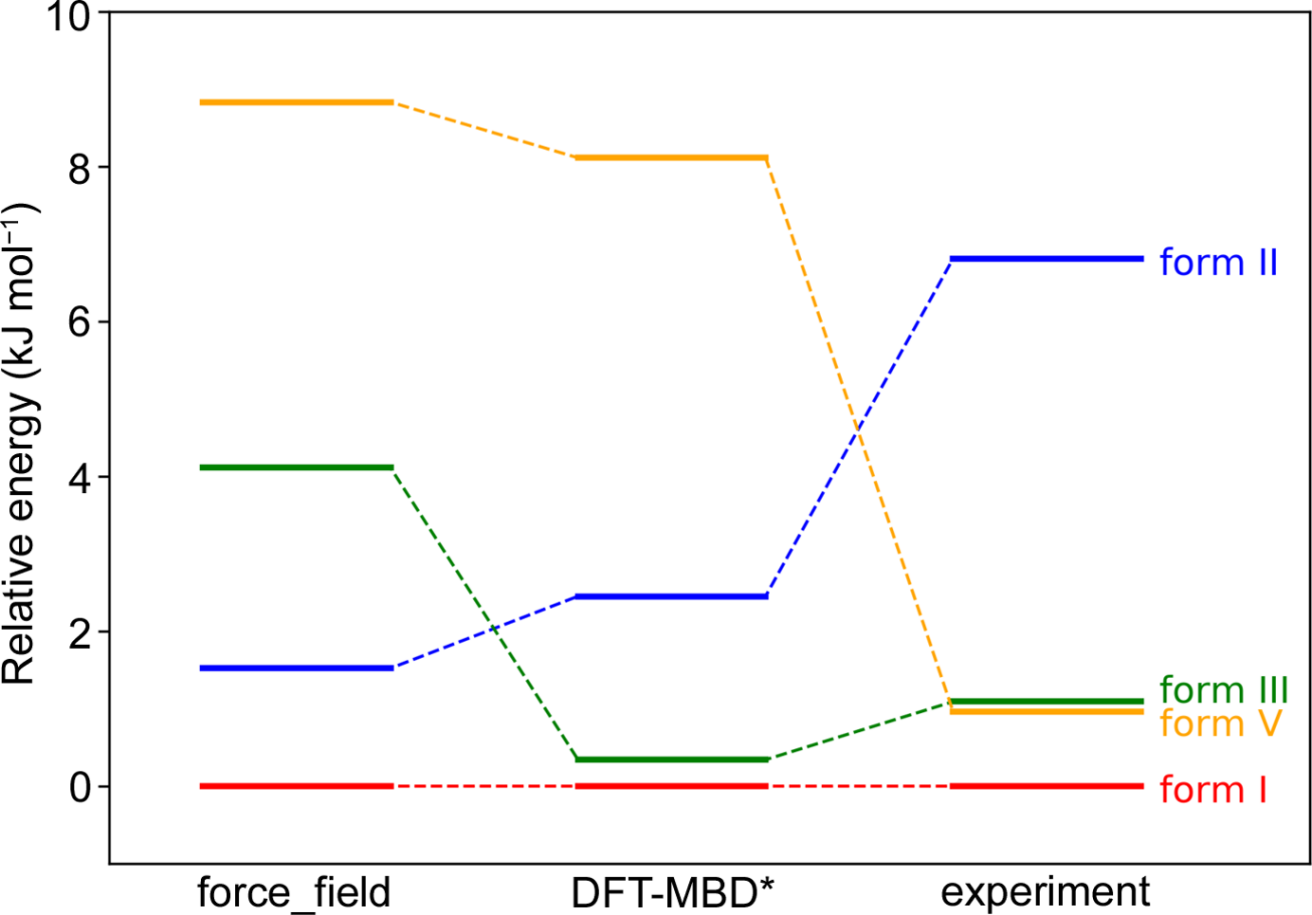
Computational and experimental energetic stability of four MLX polymorphs.

Fig. 8 ▸ features a comparison of energetic stability of four neat polymorphs of MLX. The experimental data were derived from the DSC curves, while computational ones represent either force-field relative energies (sum of intra- and intermolecular energy contributions) or DFT-MBD* relative energies after geometry optimization. Quite expectedly, in all three cases the most energetically stable is MLX-I. The experimental values for the three elusive forms indicate that MLX-III and MLX-V are around 1 kJ mol^−1^ less energetically stable than form I, while MLX-II is the least stable, with a relative energy of *ca* 7 kJ mol^−1^. These data agree with our observations made during crystallization: by far the most labile and elusive was MLX-II, which easily transformed to MLX-V during storage, whereas MLX-III and MLX-V were stable while kept under ambient conditions. These latter forms were also found to crystallize concomitantly with MLX-I from solution, though MLX-II could be obtained by mild desolvation only and never by solvent evaporation.

## 3. Conclusions

In many structural chemistry laboratories there is one preferred method for solving crystal structures of organic molecules, usually closely associated with the expertise of the researchers working on them. Our work showcases the benefit of using different crystal structure determination methods depending on the problem at hand. Still, SCXRD remains the most obvious choice in solving many structural problems of organic crystal structures, but for some demanding samples it is useful to be conscious of the benefits offered by other techniques. And though recent years have witnessed enormous progress in electron diffraction techniques, which enabled its more widespread usage in the crystallographic community, it is quite clear that, however useful the method may be, it is also not the solution to everything. Perhaps the least recognized method is that based on NMRX, sometimes considered not to be a real crystallographic method by some researchers. This is because it is often not easy to derive a viable structural model of a crystal based only on the solid-state NMR spectra. However, as shown in several recent works (Bravetti *et al.*, 2022 ▸; Dudek *et al.*, 2020*b* ▸; Hofstetter *et al.*, 2019 ▸; Widdifield *et al.*, 2020 ▸), the application of NMRX, especially in combination with CSP calculations, can add new insight into our ever-growing knowledge on organic crystal structures. Our work is a good example of the benefits of not restricting oneself to a particular crystal structure determination approach. Using three different methods – SCXRD, CSP-NMRX and microED – we were able to solve the crystal structures of three elusive forms of the anti-inflammatory drug meloxicam, a task which would not be possible if even only one of them was disregarded.

The three elusive MLX polymorphs all share the same structural unit: a dimer stabilized by NH⋯N interactions. For such interaction to form, the usually planar molecule of MLX has to be ever so slightly distorted, resulting in somewhat higher intramolecular energy of conformations present in these crystals. This in turn explains why it is so difficult to crystallize these forms from solution, in which the most energetically stable planar form is ubiquitous. We believe that this distortion from planarity is also the main reason why MLX-III could not be found in primary rigid-body CSP calculations, unless the exact experimental conformations were taken for the calculations. However, since the conformational distortion from planarity was not an issue in predicting the crystal structure of MLX-II, which in turn was not found in the flexible CSP, something more had to be at play in the case of MLX-III. This particular polymorphic form is therefore not only elusive experimentally, but also computationally. Why? For now our most probable hypothesis is because it lies in a shallow energy minimum, close to a deeper, more steep one, but further calculations are planned to fully understand this phenomenon. Revealing its causes may pave the way to a deeper understanding of the elusiveness of some of the organic crystal polymorphs.

## 4. Experimental

### 4.1. Materials

Meloxicam was purchased from ABCR and used as recieved, after evaluating its phase purity by PXRD. All crystallization experiments leading to elusive MLX polymorphic forms are described in our previous work (Jeziorna *et al.*, 2023 ▸). Briefly, MLX-II was obtained from the hexa­fluoro­iso­propanol solvate of MLX after leaving it for 6 days in an open container. MLX-III in bulk quantities was obtained from the DMSO solvate of MLX after keeping it at 130°C for 15 min. The crystals of MLX-III for SCXRD were obtained after dissolving 50 mg of MLX in a mixture of 0.5% of aqueous KOH solution (16 ml) and ethanol (16 ml), so that the molar ratio of MLX to KOH was 1:1. The mixture was heated to *ca* 50°C for 15 min using a magnetic stirrer until no visible solid was left and then filtered through a syringe filter. 80% acetic acid was then carefully added dropwise to the filtered solution with pH control, until a value between 5 and 6 was achieved. The solution remained clear and the flask with it was placed in chamber filled with toluene vapour, just above warm toluene. After a few days small crystals of two different morphologies were collected. MLX-V was obtained after the desolvation of hexa­fluoro­iso­propanol solvate at 130°C for 15 min.

### 4.2. Single-crystal X-ray diffraction experiments

Suitable crystals of MLX-III were selected and glued to the support using a silicone grease. The diffraction intensities were collected with a Rigaku XtaLAB Synergy-S diffractometer equipped with a Cu *K*α radiation source (λ = 1.5418 Å) and HyPix-6000HE hybrid photon counting detector. The total number of runs and images was based on the strategy calculation from the program *CrysAlisPro* (Rigaku, v1.171.41.123a, 2022). Molecular models of structures were obtained by the *SHELXT* (Sheldrick, 2015*a* ▸) structure solution program using intrinsic phasing with *Olex2* (Bourhis *et al.*, 2015 ▸) as the graphical interface and refined by least squares using version 2018/3 of *SHELXL* (Sheldrick, 2015*b* ▸). All non-hydrogen atoms were refined anisotropically. Hydrogen-atom positions were calculated geometrically and refined using the riding model. The structure was validated by *CheckCif* (https://checkcif.iucr.org) and deposited at the Cambridge Crystallographic Data Centre (CCDC) under the accession code 2293153.

### 4.3. Powder X-ray diffraction experiments

The PXRD patterns were registered on a Panalytical Empyrean powder X-ray diffractometer, in horizontal Bragg–Brentano mode, using copper radiation (λ = 1.5419 Å) and zero-background holders. The 2θ range in each case was 3–45°, and the hardware setup was as follows: 0.02 rad Soller slits, fixed 4 mm mask, 1/4° anti-scatter slit and 1/16° divergence slit for the incident beam; large 0.02 rad Soller slits and 7.5 mm anti-scatter slit for the divergent beam. The samples were spun with a rotation time of 1 Hz to ensure proper sample averaging and a 3D PIXcel detector with all 255 active channels was used. Typically, the step size was equal to 0.0131°, the time per step was set to 25 s and three scans were acquired.

### 4.4. Solid-state NMR spectroscopy

All NMR experiments were acquired using a Bruker Avance III 600 spectrometer, which operates at 150.92, 60.82 and 600.15 MHz frequencies for ^13^C, ^15^N and ^1^H, respectively. For the ^13^C and ^15^N CPMAS spectra, a 13 333 Hz rotation; 90° pulse durations of 3.19 and 2.50 µs, respectively; a relaxation delay of 6.89 s; and a 2 ms contact time were used. The experiments were performed with a 4 mm Bruker CP-MAS ^1^H/BB probe head. The spectra were referenced to adamantane (38.48 p.p.m. for ^13^C) and α-glycine (32.4 p.p.m. for ^15^N) used as secondary reference materials. The 2D inv-^1^H–^13^C HETCOR and ^1^H–^1^H SQ–DQ BaBa experiments were done on a 1.3 mm Bruker probe head, using a sample rotation of 55 555 Hz and a relaxation delay of 10.5 s. For the HETCOR experiments, a pulse sequence proposed by Pruski was used (Mao *et al.*, 2009 ▸; Althaus *et al.*, 2014 ▸), with the first and second contact times set at 2 ms and 50 µs, respectively, to observe direct C—H connectivities, and at 3 ms, to observe longer range correlations. The experiments were carried out with a low-power swept-frequency two-pulse phase modulation decoupling sequence (Chandran *et al.*, 2008 ▸).

### 4.5. Electron diffraction

**Figure 9:**
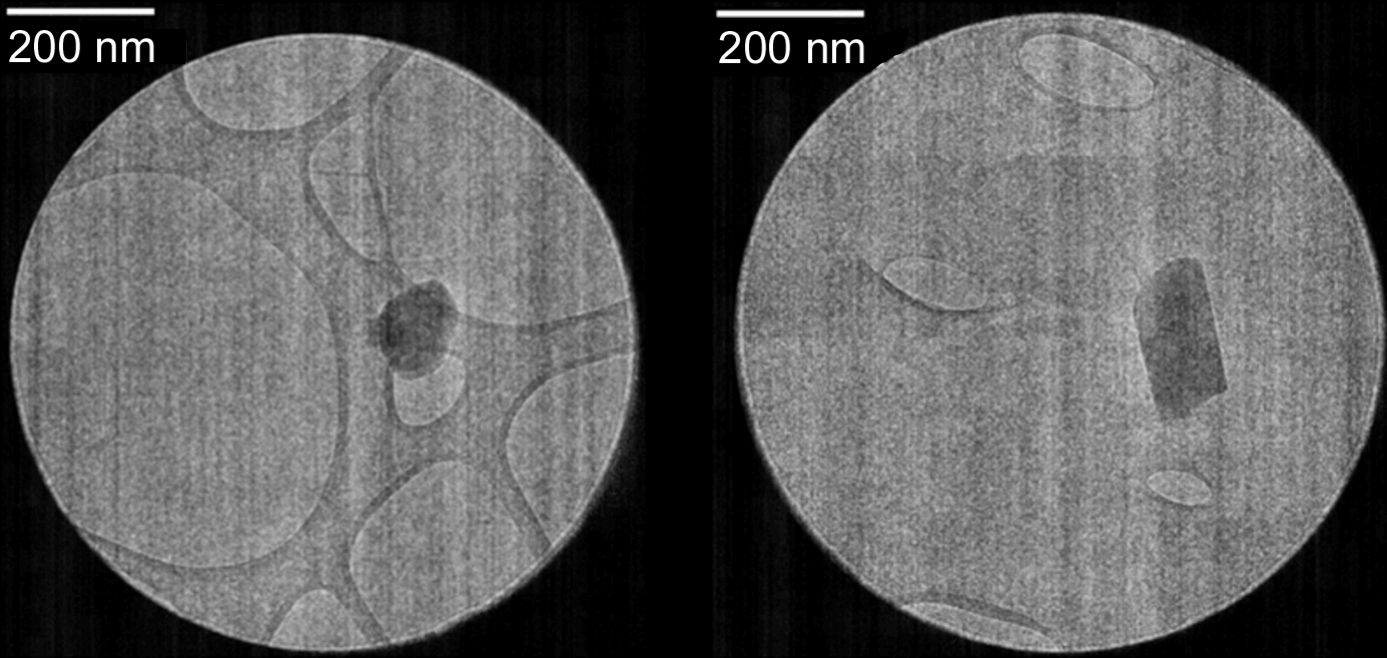
TEM images of an MLX-V microcrystal.

A small amount of the sample was first gently crushed in a mortar and pestle to reduce the crystal size. Grids for microED data collection were prepared by directly applying a pinch of powdered crystals to a freshly glow-discharged lacey carbon 200 mesh Cu grid. Following that, the grids were clipped at room temperature and transferred to the microscope for data collection. The grids were then cooled while the microscope was cooling under the vacuum. A Thermo Fisher Scientific Glacios cryo transmission electron microscope (TEM) equipped with a field emission gun operated at 200 kV and a stage holder temperature of 80 K were used for single-crystal data collection (Fig. 9 ▸). The microscope was equipped with a Thermo Fisher Scientific CETA-D detector, an autoloader with twelve grid holders and the *EPU-D* software for automated data collection. A 50 µm condenser aperture, spot size 11 and gun lens 8 were set and diffraction datasets were collected under parallel illumination conditions with a very low dose (3.6 e Å^−2^). The crystal was continuously rotated from −60 to +60° under the paralleled beam. The microscope was set in a diffraction mode and the camera collected continuously in a rolling shutter mode with a hardware binning of 2 and an exposure time of 0.5 s. The images collected were saved in SMV format built into the *EPU-D* software.

### 4.6. Data processing and refinement details

Frames were indexed and integrated in *XDS* (Kabsch, 2010 ▸) and the intensities were converted to *SHELX* format using *XPREP*. The final structure factors are the results of merging data from two crystals using *XSCALE* to achieve a completeness of 83% for the triclinic crystal structure. The structures were solved in *SHELXT* (Sheldrick, 2015*a* ▸). All the structural refinements were performed in *Olex2* (Bourhis *et al.*, 2015 ▸) using *olex2.refine* in the kinematical diffraction theory approach. In the refinement, the following weighting scheme was applied: *w* = 1/[σ^2^(*F*_o_^2^) + (0.2*P*)^2^], where *P* = (*F*_o_^2^ + 2F_c_^2^)/3. The structure was deposited at the CCDC under the accession code 2335502.

### 4.7. DSC and TGA analysis

DSC measurements were performed on a DSC 2500 (TA Instruments) calibrated on indium. The measurements were performed in hermetically sealed aluminium pans at a heating rate of 5°C min^−1^ and a nitro­gen flow of 50 ml min^−1^. TGA measurements were performed with a TGA 5500 (TA Instruments) using high-temperature platinum pans and a 5°C min^−1^ heating rate.

### 4.8. NMR calculations for CSP-derived structural models

The lowest-energy crystal structures of MLX were retrieved from our previous CSP calculations and geometry optimized using a *CASTEP* code (Clark *et al.*, 2005 ▸) and PBE-MBD* functional (Kronik & Tkatchenko, 2014 ▸), with all parameters allowed to vary. An energy cut-off of 1000 eV and a *k*-point separation of 0.07 Å^−1^ were used. For all unique structures, the NMR shielding constants were calculated with a GIPAW approach (Yates *et al.*, 2007 ▸) and recalculated to chemical shifts using the δ_calc_ = (σ_calc_ − *b*)/*m* linear equation established for each dataset by plotting assigned experimental chemical shifts against theoretical shielding constants (*b* – intercept, *m* – slope). The differences in the calculated and theoretical chemical shifts are expressed as RMSD values obtained from the comparison of experimental and theoretical chemical shifts, and are given in p.p.m.

### 4.9. *CASTEP* calculations for MLX-III and MLX-V

The experimentally determined crystal structures of MLX-III and MLX-V were geometry optimized under periodic boundary conditions with a *CASTEP* code using the parameters as described above for MLX-II.

### 4.10. CSP calculations for MLX-III

The *Z*′ = 2 crystal structure generation in the pursuit of MLX-III was carried out using *Global Lattice Energy Explorer* (Case *et al.*, 2016 ▸), which is based on a quasi-random search. In the search up to 100 000 successfully geometry minimized crystal structures were generated in *P*1, which is the correct SG for MLX-III. The first geometry minimization was done with respect to intermolecular interactions in *DMACRYS* (Price *et al.*, 2010 ▸), employing the FIT repulsion-dispersion potential (Coombes *et al.*, 1996 ▸). The *GDMA 2.2.11* software (Stone, 2005 ▸) was used to generate atom-centred multipoles up to rank 4 from the electron densities calculated with the *Gaussian16* software (Frisch *et al.*, 2016 ▸) at the B3LYP-GD3BJ/6-311G(d,p) level of theory (Becke, 1993 ▸; Grimme *et al.*, 2011 ▸). The cut-off value for van der Waals interactions was set to 25 Å. The structures were then evaluated for duplicates on the basis of similarity of their PXRD patterns, as well as density and energy data with the cut-off values set to 0.02 g cm^−3^ and 0.1 kJ mol^−1^, respectively. Finally, the structures were evaluated against the experimental crystal structure of MLX-III using the *Crystal Packing Similarity Tool* (Chisholm & Motherwell, 2005 ▸).

In the rigid CSP search, we tested several different conformations of MLX in the CSP searches in different combinations. The default approach is to use viable gas-phase minima, which in our case included only the lowest-energy conformation of MLX, m3. Apart from this we also tested several conformations slightly distorted from m3, as well as molecular conformations from the geometry optimized MLX-III structure in different combinations. All of the tested arrangements, together with their difference to the arrangements found in the experimental crystal structure are featured in Table S10.

In the flexible search, CSP calculations were carried out using the code *CrystalPredictor* version 2.4.3 (Habgood *et al.*, 2015 ▸) using a default workflow. The flexible conformational degrees of freedom were determined based on the changes in intramolecular energy values arising from ±15° perturbations applied to those torsional angles that were identified as potentially flexible by the values of second derivatives at the gas-phase conformational minimum. Isolated-molecule QM calculations were performed in *Gaussian 09* at the PBE0 level of theory using the 6-311G(d,p) basis set. Starting with an initial uniform LAM grid, the adaptive LAM algorithm was run (Sugden *et al.*, 2016 ▸) until convergence was achieved, with the convergence criterion Δ* equal to 5 kJ mol^−1^; this resulted in 3 LAMs. The set of parameters referred to as the ‘FIT potential’ (Coombes *et al.*, 1996 ▸; Beyer & Price, 2000 ▸; Williams & Cox, 1984 ▸; Cox *et al.*, 1981 ▸; Motherwell *et al.*, 2002 ▸) was used to describe the exchange-repulsion and dispersion interactions. In the global search space, 1 000 000 structure minimizations were performed, sampling the 59 most common SGs in *Z*′ = 2.

After the *CrystalPredictor* calculations were completed, a final clustering of generated structures was carried out with the *COMPACK* algorithm (Chisholm & Motherwell, 2005 ▸).

MLX-I and MLX-III were found in the resulting set of structures (the global minimum structure has an RMSD_15_ = 0.457 Å match with SEDZOQ), and the rank 41 structure matches form MLX-III with RMSD_15_ = 0.364 Å (see Fig. S8 for structure comparison), but form MLX-II was not observed in the final set of structures. This suggests that a more comprehensive search than the standard workflow would be required to correctly find the structure, perhaps making use of enhanced sampling methods minimum (Francia *et al.*, 2021 ▸), but this was considered out of scope, since MLX-III was explicitly being searched for.

### 4.11. Gas-phase calculations for tautomeric forms of MLX

Possible tautomeric forms of MLX were prepared from the initial geometry taken from the crystal structure of MLX-I and subjected to geometry optimization in the gas phase using the *Gaussian16* software (Frisch *et al.*, 2016 ▸) and the B3LYP-GD3BJ/6-311G(d,p) level of theory (Becke, 1993 ▸; Grimme *et al.*, 2011 ▸).

## Supplementary Material

Crystal structure: contains datablock(s) I. DOI: 10.1107/S2052252524011898/ed5031sup1.cif

Structure factors: contains datablock(s) I. DOI: 10.1107/S2052252524011898/ed5031sup2.hkl

CIF for MLX-V, determined by microED. DOI: 10.1107/S2052252524011898/ed5031sup3.txt

CIF for the CSP-generated structure of MLX-II. DOI: 10.1107/S2052252524011898/ed5031sup4.txt

Structural data for MLX-III and MLX-V, and other data, including PXRD, solid-state NMR, DSC and TGA, and computational data. DOI: 10.1107/S2052252524011898/ed5031sup5.pdf

CCDC references: 2293153, 2335502

## Figures and Tables

**Table 1:** Crystal structure parameters for MLX-III

	MLX-III
CCDC code	2293153
Chemical formula	C_14_H_13_N_3_O_4_S_2_
Formula weight	702.79
Crystal system	Triclinic
Space group	*P* 1
Temperature (K)	100.00 (10)
*a* (Å)	11.2439 (2)
*b* (Å)	11.2772 (2)
*c* (Å)	13.4027 (2)
α (°)	65.901 (2)
β (°)	87.254 (1)
γ (°)	79.439 (1)
*V* (Å^3^)	1524.37 (5)
*Z*	4
*Z*′	2
*d*_calc_ (g cm^−3^)	1.531
Crystal dimensions (mm)	0.13 × 0.05 × 0.04
Radiation type	Cu *K*α
μ (mm^−1^)	3.396

**Table 2:** Data collection, reduction and refinement statistics for MLX-V

	MLX-V
CCDC code	2335502
Chemical formula	C_14_ H_13_N_3_O_4_S_2_
Tilt angle/tilt speed (°)	0.5/1
Detector distance (mm)	644.72
Temperature (K)	80
Accelerating voltage (kV)	200
Wavelength (Å)	0.02508

Data reduction	
Space group	*P* 1
Unit cell *a*, *b*, *c* (Å)	13.8, 15.5, 15.5
Angles α, β, γ (°)	63.5, 85.8, 85.6
Volume (Å^3^)	2955.5
Resolution (Å)	0.67
Total reflections	52088
Unique reflections	16910
Completeness (%)	83.3

Kinematical refinement	
No. of measured, independent and observed [*I* > 2σ(*I*)] reflections	52088, 16910, 3817
Parameters	831
*R*1 [*I* > 2σ(*I*)]	0.1708
*wR*2 [*I* > 2σ(*I*)]	0.3961
*R*1 (all data)	0.4016
*wR*2 (all data)	0.5174
GooF	0.9391
Residual potential max./min. (Å^−2^)	1.48/−1.57
