# Supplementary material for: SCXRD, CSP-NMRX and microED in the quest for three elusive polymorphs of meloxicam
Source: IUCrJ. 2025 Jan 1;12(Pt 1):109–22. doi: 10.1107/S2052252524011898 (PMC11707701; doi:10.1107/S2052252524011898)
Supplement: Supplementary file 5 [file m-12-00109-sup5.pdf]

IUCrJ

Volume 12 (2025)

**Supporting information for article:**

# SCXRD, CSP-NMRX and microED in the quest for three elusive polymorphs of meloxicam

Agata Jeziorna, Maura Malinska, Isaac Sugden, Piotr Paluch, Rafał Dolot and Marta Dudek

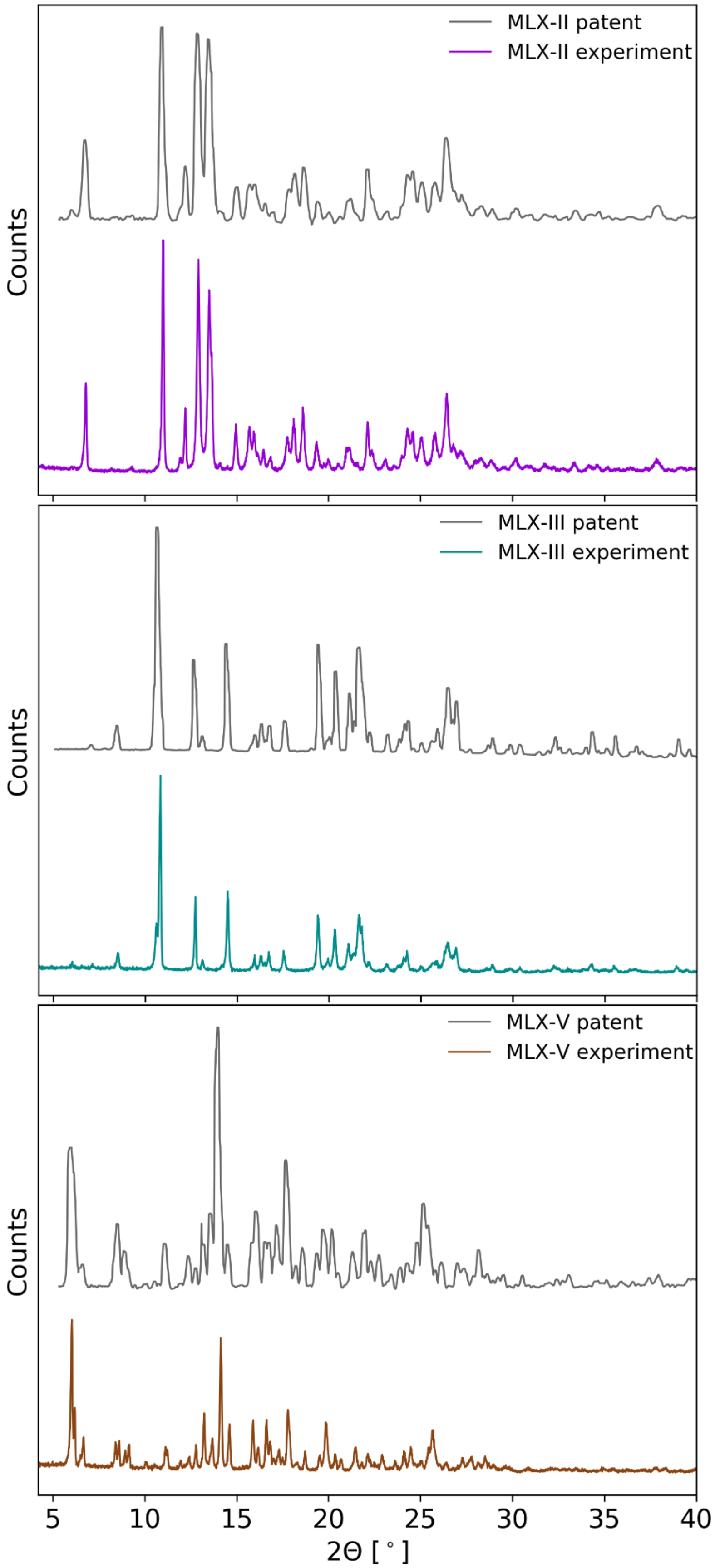


**Figure S1.** Comparison of PXRD patterns for MLX-II, III and V crystal forms disclosed in the original MLX patent [1] with the experimental patterns obtained for crystal forms examined in this work.

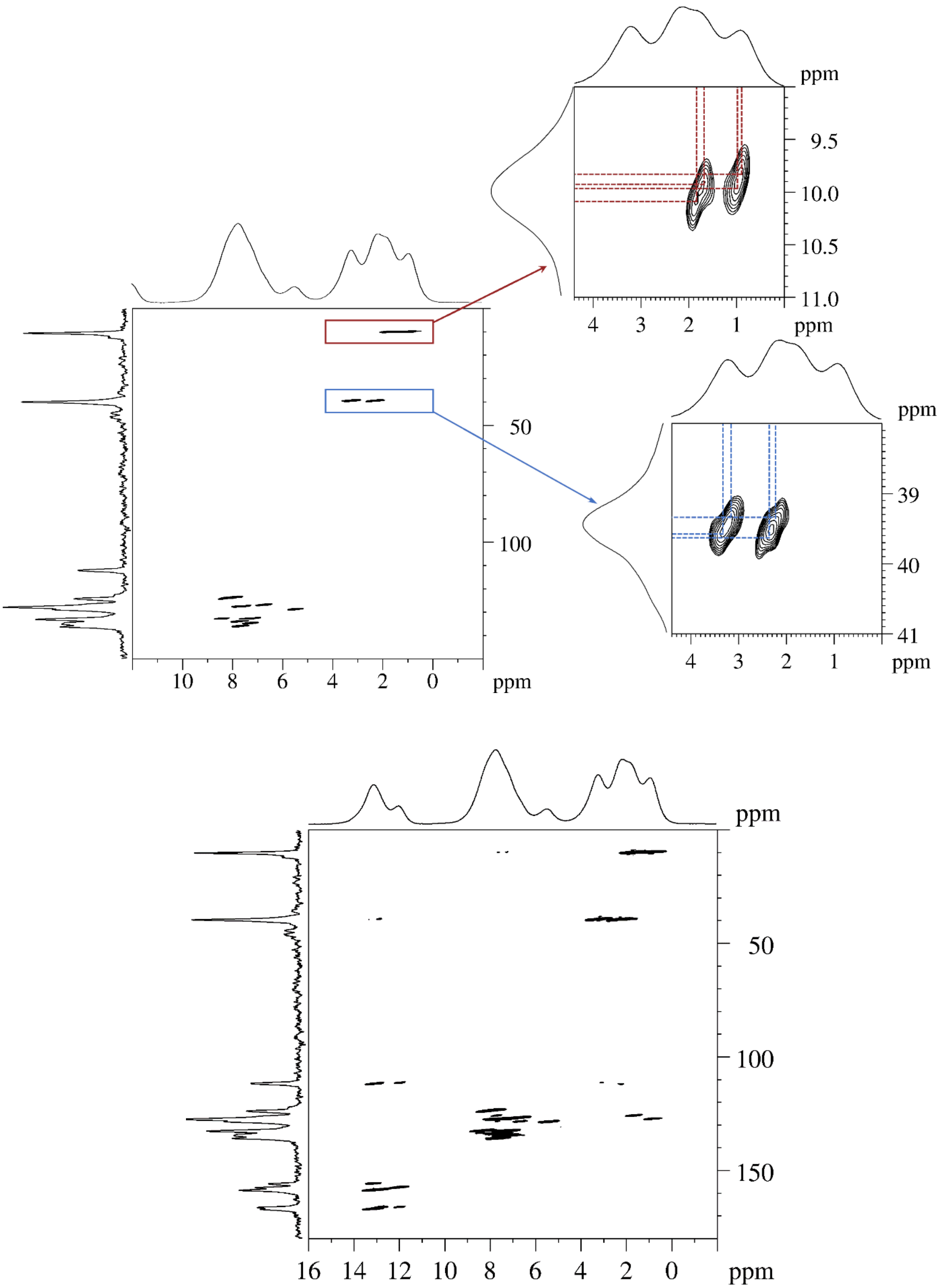


**Figure S2.** *Inv*-$^{1}$H-$^{13}$C HETCOR NMR spectra ($\nu_R$ = 55.55kHz) registered for MLX-V. Upper panel shows spectrum with a short second contact time of 100 µs, revealing direct $^{1}$H-$^{13}$C pairs, together with expansions on methyl group signals regions. Lower panel shows spectrum with a long second contact time of 2 ms, showing further $^{1}$H-$^{13}$C connectivities.

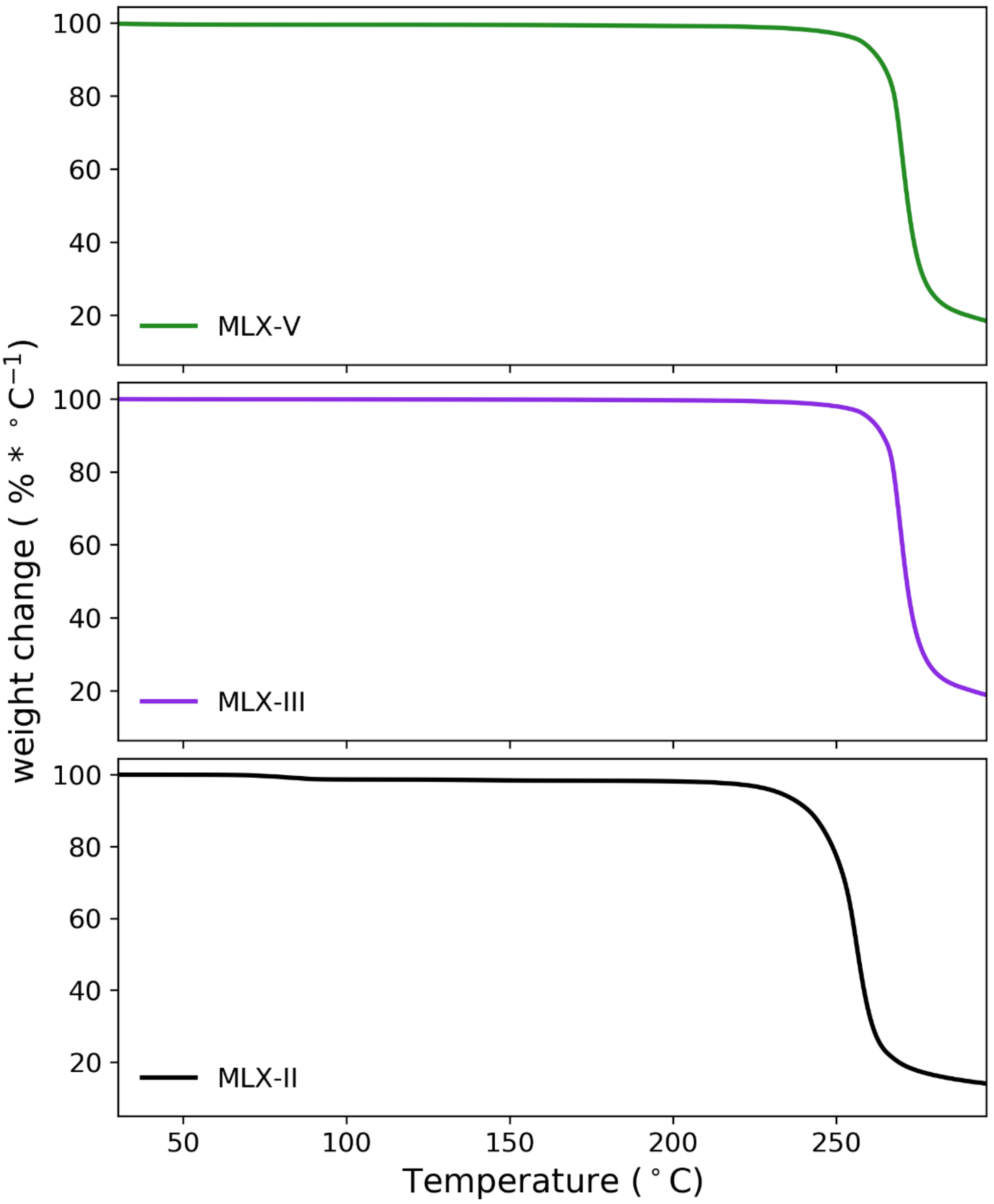


**Figure S3.** TGA plots for MLX-II, III and V.

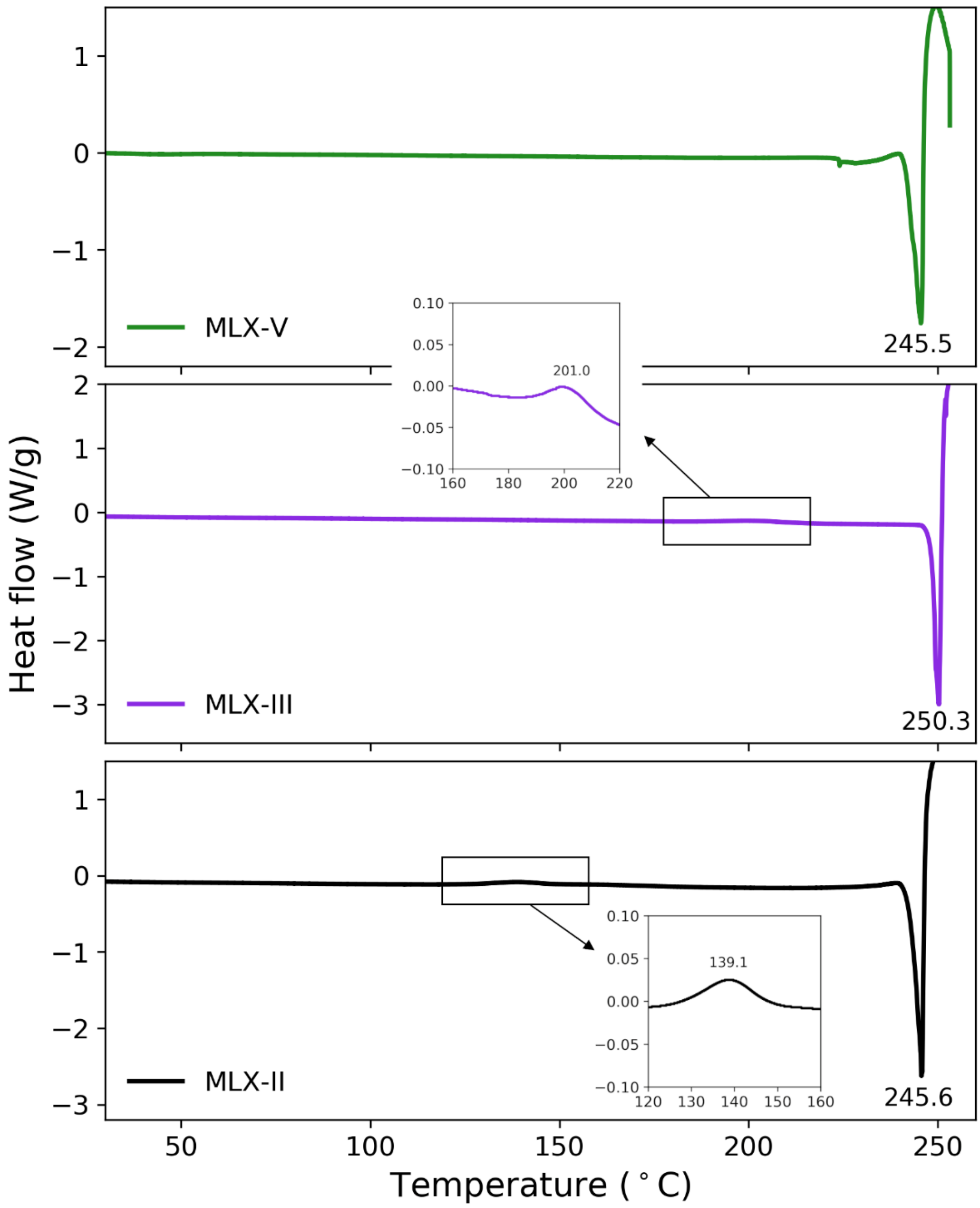


**Figure S4.** DSC plots for MLX-II, III and V. The inlets show phase transitions of MLX-II to MLX-V at ca. 139°C (the lowest panel) and of MLX-III to MLX-I at ca. 201°C (middle panel).

**Table S1.** $^{1}$H, $^{13}$C and $^{15}$N chemical shifts for MLX-I and MLX-II (in ppm).

| | MLX-I | | MLX-II-A | | MLX-II-B | |
|---|---|---|---|---|---|---|
| | δ($^{13}$C) | δ($^{1}$H) | δ($^{13}$C) | δ($^{1}$H) | δ($^{13}$C) | δ($^{1}$H) |
| C-1 | 133.9 | | 133.2 | | 135.4 | |
| C-2 | 127.0 | 7.2 | 125.1 | 7.5 | 126.4 | 7.0 |
| C-3 | 134.3 | 7.6 | 133.0 | 7.4 | 132.3 | 6.9 |
| C-4 | 131.3 | 6.5 | 133.4 | 7.1 | 133.0 | 6.8 |
| C-5 | 125.2 | 6.5 | 126.4 | 6.8 | 127.6 | 6.7 |
| C-6 | 128.5 | | 128.4 | | 128.1 | |
| C-7 | 160.1 | 12.7 | 160.2 | 12.7 | 159.9 | 13.0 |
| C-8 | 110.1 | | 112.4 | | 111.5 | |
| C-9(N-Me) | 42.3 | 2.9 | 38.7 | 2.9 | 38.7 | 2.7 |
| C-10 | 165.9 | | 167.0 | | 167.0 | |
| NH | | 9.2 | | 13.2 | | 13.2 |
| C-11 | 154.4 | | 158.4 | | 158.4 | |
| C-12(CH) | 128.6 | 6.1 | 135.6 | 7.5 | 136.2 | 7.1 |
| C-13 | 137.6 | | 133.6 | | 135.0 | |
| C-14(Me) | 13.0 | 2.0 | 11.0 | 1.2 | 11.5 | 0.9 |
| | δ($^{15}$N) | | δ($^{15}$N) | | δ($^{15}$N) | |
| N1 | 83.0 | | 81.1 | | 81.1 | |
| N2 | 131.0 | | 134.1 | | 134.1 | |
| N3 | 280.1 | | 267.4 | | 266.9 | |

**Table S2.** Numerical values for $^{1}$H and $^{13}$C RMSD values presented graphically in Figure 4. The relative DFT-MBD* energies are given in respect to the energy of the global minimum Z' = 1 structure. In bold two structures considered in the text are marked.

| Structure name | Relative energy (kJ/mol of molecules) | $^{1}$H RMSD (ppm) | $^{13}$C RMSD (ppm) |
|---|---|---|---|
| 1_14 | 2.81 | 1.46 | 2.25 |
| 6_15 | 2.84 | 1.46 | 2.31 |
| 1_15 | 3.63 | 1.30 | 2.24 |
| 3_15 | 4.52 | 1.50 | 2.19 |
| 3_14 | 4.81 | 1.35 | 2.2 |
| **6_14** | **5.23** | **0.32** | **1.68** |
| 5_14 | 5.67 | 1.55 | 2.45 |
| 31_2 | 5.94 | 0.74 | 4.35 |
| 16_14 | 6.47 | 0.87 | 2.12 |
| 2_2 | 7.14 | 1.53 | 2.38 |
| **9_14** | **7.88** | **0.33** | **1.58** |
| 2_15 | 8.31 | 1.52 | 2.23 |
| 13_14 | 9.91 | 1.73 | 2.42 |
| 33_14 | 10.17 | 1.47 | 2.69 |
| 2_14 | 10.37 | 1.19 | 2.33 |
| 36_2 | 10.55 | 0.64 | 4.22 |
| 21_14 | 10.84 | 1.29 | 2.36 |
| 11_15 | 11.64 | 1.51 | 2.43 |
| 1_4 | 11.83 | 1.36 | 2.41 |
| 37_14 | 12.05 | 1.16 | 2.57 |
| 42_14 | 12.24 | 1.59 | 2.22 |
| 8_14 | 12.31 | 1.34 | 2.31 |
| 20_14 | 12.60 | 1.21 | 2.34 |
| 7_2 | 12.96 | 1.39 | 2.14 |
| 1_33 | 13.05 | 2.29 | 2.31 |
| 1_2 | 13.10 | 1.19 | 2.69 |
| 17_14 | 13.27 | 2.12 | 2.26 |
| 4_14 | 14.22 | 1.36 | 2.46 |
| 46_14 | 14.23 | 1.27 | 2.72 |
| 5_2 | 14.61 | 1.04 | 3.51 |

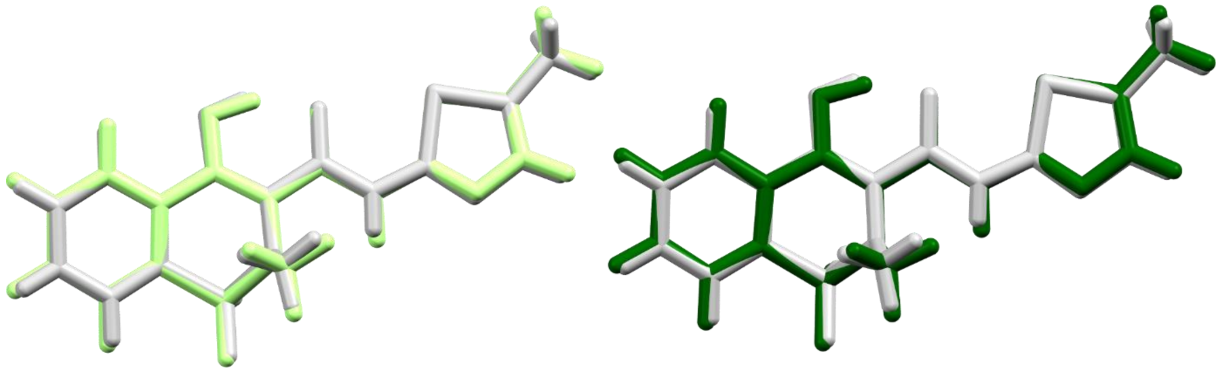

**Figure S5.** Molecular overlay of two symmetry-independent MLX conformations building MLX-II (light green and dark green) with a conformation building MLX-I (grey).

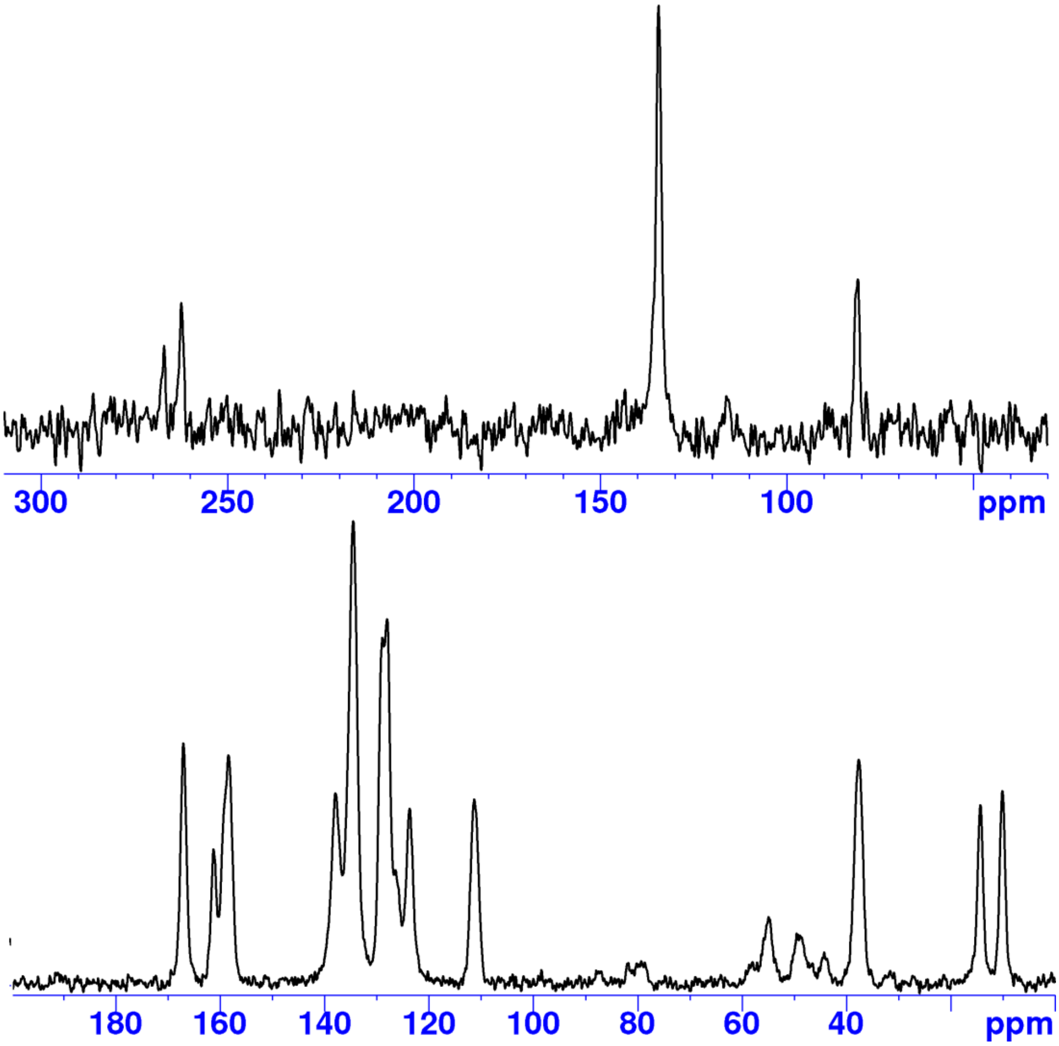


**Figure S6.** $^{15}N$ (upper) and $^{13}C$ (lower) CPMAS NMR spectra ($\nu_R$ = 13.333 kHz) registered for MLX-III.

**Structural data from scXRD for MLX-III**

**Table S3.** Fractional Atomic Coordinates (×10$^4$) and Equivalent Isotropic Displacement Parameters (Å$^2$×10$^3$) for **MLX III**. $U_{eq}$ is defined as 1/3 of the trace of the orthogonalized $U_{ij}$.

| Atom | x | y | z | $U_{eq}$ |
|---|---|---|---|---|
| S1 | 6013.4(4) | 7802.1(5) | 4612.3(4) | 18.73(12) |
| S2 | 10768.1(4) | 5183.8(5) | 2395.5(4) | 18.76(12) |
| O1 | 6078.5(12) | 8853.6(14) | 3562.2(11) | 22.2(3) |
| O2 | 4878.4(12) | 7393.2(15) | 4969.1(13) | 25.6(3) |
| O3 | 10127.1(12) | 6253.7(14) | 3798.2(12) | 23.2(3) |
| O7 | 9794.6(12) | 7669.3(15) | 4884.1(13) | 25.4(3) |
| N1 | 6997.1(14) | 6521.7(16) | 4625.4(13) | 18.2(3) |
| N2 | 8550.8(14) | 5601.9(16) | 3292.2(14) | 19.1(3) |
| N3 | 8779.4(14) | 4518.0(16) | 2106.9(14) | 20.1(3) |
| C1 | 6634.6(17) | 8210.6(19) | 5585.6(16) | 18.8(4) |
| C2 | 5900.3(18) | 8756(2) | 6204.2(17) | 21.7(4) |
| C3 | 6432.7(19) | 9197(2) | 6874.4(17) | 23.6(4) |
| C4 | 7684.8(19) | 9075(2) | 6918.8(18) | 25.5(4) |
| C5 | 8413.8(18) | 8510(2) | 6308.0(18) | 23.4(4) |
| C6 | 7900.5(17) | 8062.5(19) | 5626.1(16) | 19.6(4) |
| C7 | 8650.4(17) | 7458(2) | 4977.1(16) | 19.9(4) |
| C8 | 8226.0(17) | 6747.7(19) | 4497.4(16) | 18.4(4) |
| C9 | 6826.2(19) | 5202(2) | 5446.4(18) | 25.2(4) |
| C10 | 9022.0(18) | 6195.0(19) | 3845.8(16) | 19.7(4) |
| C11 | 9249.1(17) | 5081.1(19) | 2631.4(16) | 18.5(4) |
| C12 | 9648.0(17) | 4156.4(19) | 1463.2(16) | 19.9(4) |
| C13 | 10764.4(17) | 4420.4(19) | 1508.1(16) | 19.5(4) |
| C14 | 11881.0(18) | 4105(2) | 943.3(18) | 23.4(4) |
| | | | | |
| S21 | 8852.8(4) | 1336.8(5) | 1250.2(4) | 19.96(12) |
| S22 | 4038.1(4) | 5765.1(5) | 1582.8(4) | 19.74(12) |
| O21 | 8788.3(13) | 2392.8(14) | 183.6(12) | 23.7(3) |
| O22 | 9984.9(12) | 817.9(15) | 1848.2(14) | 28.6(3) |
| O23 | 4753.9(12) | 3586.5(14) | 1240.3(12) | 23.3(3) |

| Atom | x | y | z | $U_{eq}$ |
|---|---|---|---|---|
| O27 | 5095.8(12) | 1633.8(14) | 735.2(12) | 22.2(3) |
| N21 | 7852.7(14) | 1845.4(16) | 1993.9(14) | 19.3(3) |
| N22 | 6270.9(14) | 4124.0(16) | 1933.5(14) | 19.6(3) |
| N23 | 5958.1(15) | 5858.5(17) | 2513.9(14) | 21.5(4) |
| C21 | 8266.9(18) | 59.6(19) | 1131.4(16) | 19.7(4) |
| C22 | 9027.1(18) | -992(2) | 1022.5(17) | 22.0(4) |
| C23 | 8521.3(18) | -1887.1(19) | 786.0(17) | 21.6(4) |
| C24 | 7267.2(18) | -1735(2) | 698.7(17) | 22.0(4) |
| C25 | 6512.7(18) | -695(2) | 829.9(16) | 20.7(4) |
| C26 | 7000.1(17) | 233.7(19) | 1042.4(15) | 18.0(4) |
| C27 | 6229.8(17) | 1383(2) | 1130.2(16) | 18.9(4) |
| C28 | 6637.5(17) | 2162.9(19) | 1549.2(16) | 18.6(4) |
| C29 | 7967(2) | 1082(2) | 3198.1(17) | 27.3(5) |
| C30 | 5833.9(17) | 3331(2) | 1559.2(16) | 19.7(4) |
| C31 | 5537.4(17) | 5196(2) | 2047.8(16) | 19.3(4) |
| C32 | 5055.5(18) | 6878(2) | 2507.7(16) | 20.7(4) |
| C33 | 3968.8(18) | 6994.8(19) | 2054.5(16) | 20.2(4) |
| C34 | 2827.5(19) | 7959(2) | 1981.5(18) | 25.5(4) |

**Table S4.** Anisotropic Displacement Parameters (×10[4]) **MLX III**. The anisotropic displacement factor exponent takes the form: $-2\pi^2[h^2a^{*2} \times U_{11} + \ldots + 2hka^* \times b^* \times U_{12}]$

| Atom | $U_{11}$ | $U_{22}$ | $U_{33}$ | $U_{23}$ | $U_{13}$ | $U_{12}$ |
|---|---|---|---|---|---|---|
| S1 | 14.1(2) | 23.5(2) | 20.8(2) | -11.3(2) | -1.93(17) | -2.57(17) |
| S2 | 13.2(2) | 23.3(2) | 22.8(2) | -12.1(2) | -1.48(17) | -3.31(17) |
| O1 | 20.6(7) | 23.9(7) | 22.1(7) | -10.2(6) | -4.8(6) | -0.6(6) |
| O2 | 15.7(7) | 35.9(8) | 31.8(8) | -19.3(7) | 1.5(6) | -7.5(6) |
| O3 | 14.7(7) | 30.8(8) | 28.6(8) | -16.3(7) | -0.5(6) | -4.4(6) |
| O7 | 14.7(7) | 35.0(8) | 34.8(8) | -21.6(7) | 1.8(6) | -7.0(6) |
| N1 | 15.3(8) | 20.2(8) | 20.4(8) | -8.7(7) | -0.3(6) | -5.0(6) |
| N2 | 13.5(7) | 23.5(8) | 22.2(8) | -11.0(7) | 0.6(6) | -4.0(6) |
| N3 | 16.7(8) | 22.9(8) | 22.6(9) | -10.4(7) | 0.5(6) | -5.6(6) |
| C1 | 19.4(9) | 19.4(9) | 18.1(9) | -7.9(8) | -2.7(7) | -3.5(7) |

| Atom | $U_{11}$ | $U_{22}$ | $U_{33}$ | $U_{23}$ | $U_{13}$ | $U_{12}$ |
|---|---|---|---|---|---|---|
| C2 | 18.5(9) | 23.9(10) | 22.0(10) | -9.2(8) | -0.6(8) | -2.1(8) |
| C3 | 24.2(10) | 25.5(10) | 21.2(10) | -11.4(9) | -2.5(8) | 0.4(8) |
| C4 | 25.5(10) | 26.6(11) | 27.5(11) | -14.8(9) | -7.4(9) | -0.8(8) |
| C5 | 18.5(9) | 24.8(10) | 28.0(11) | -12.0(9) | -4.8(8) | -2.1(8) |
| C6 | 17.6(9) | 20.0(9) | 20.2(10) | -7.4(8) | -2.1(8) | -1.8(7) |
| C7 | 15.6(9) | 21.9(10) | 20.0(10) | -6.2(8) | -2.3(7) | -2.8(7) |
| C8 | 14.5(9) | 21.2(9) | 18.8(9) | -7.1(8) | -1.1(7) | -3.4(7) |
| C9 | 23.9(10) | 23.9(10) | 25.3(11) | -5.9(9) | 1.4(8) | -8.3(8) |
| C10 | 19.7(9) | 18.0(9) | 19.3(10) | -5.9(8) | -2.0(7) | -2.3(7) |
| C11 | 16.3(9) | 18.0(9) | 20.4(10) | -7.0(8) | -1.8(7) | -2.2(7) |
| C12 | 19.4(9) | 20.4(9) | 21.1(10) | -9.6(8) | 0.2(8) | -3.6(7) |
| C13 | 17.9(9) | 18.9(9) | 22.3(10) | -9.5(8) | -1.8(8) | -1.7(7) |
| C14 | 16.0(9) | 29.4(11) | 29.2(11) | -16.4(9) | -0.5(8) | -3.5(8) |
| | | | | | | |
| S21 | 13.7(2) | 21.8(2) | 27.2(3) | -12.0(2) | -0.81(18) | -4.53(17) |
| S22 | 15.7(2) | 24.0(2) | 23.2(2) | -12.9(2) | -1.03(18) | -4.05(18) |
| O21 | 21.1(7) | 24.7(7) | 29.0(8) | -13.0(6) | 5.7(6) | -9.0(6) |
| O22 | 15.1(7) | 31.3(8) | 44.0(9) | -19.8(7) | -6.3(6) | -2.4(6) |
| O23 | 14.2(6) | 29.1(8) | 32.1(8) | -17.8(7) | -0.8(6) | -3.7(6) |
| O27 | 14.8(6) | 26.4(7) | 30.0(8) | -16.3(6) | -2.3(6) | -2.7(5) |
| N21 | 14.6(8) | 23.2(8) | 22.0(8) | -10.1(7) | -2.2(6) | -4.8(6) |
| N22 | 13.6(7) | 24.3(8) | 24.6(9) | -13.1(7) | 0.5(6) | -4.5(6) |
| N23 | 19.5(8) | 25.1(9) | 22.7(9) | -11.5(7) | 0.2(7) | -6.7(7) |
| C21 | 18.6(9) | 20.3(9) | 20.9(10) | -7.8(8) | -2.0(8) | -5.5(7) |
| C22 | 17.4(9) | 22.1(10) | 24.4(10) | -7.2(8) | -1.7(8) | -3.3(8) |
| C23 | 22.2(10) | 17.3(9) | 23.9(10) | -7.5(8) | -0.8(8) | -2.0(8) |
| C24 | 22.8(10) | 21.1(10) | 23.2(10) | -8.8(8) | -1.8(8) | -6.7(8) |
| C25 | 17.9(9) | 24.3(10) | 20.0(10) | -8.1(8) | -1.0(8) | -6.3(8) |
| C26 | 18.0(9) | 20.2(9) | 14.8(9) | -5.6(8) | -1.4(7) | -4.3(7) |
| C27 | 15.0(9) | 23.2(10) | 18.0(9) | -7.3(8) | -0.2(7) | -4.9(7) |
| C28 | 14.8(9) | 22.0(10) | 19.4(9) | -8.0(8) | -0.1(7) | -5.0(7) |
| C29 | 25.7(11) | 32.4(12) | 22.6(11) | -8.1(9) | -5.4(8) | -8.0(9) |

| Atom | $U_{11}$ | $U_{22}$ | $U_{33}$ | $U_{23}$ | $U_{13}$ | $U_{12}$ |
|---|---|---|---|---|---|---|
| C30 | 16.9(9) | 23.9(10) | 19.6(10) | -9.0(8) | 1.9(7) | -6.7(8) |
| C31 | 16.5(9) | 23.3(10) | 19.3(9) | -8.8(8) | 0.9(7) | -6.4(7) |
| C32 | 22.8(10) | 21.1(10) | 20.9(10) | -10.6(8) | 0.3(8) | -5.4(8) |
| C33 | 22.7(10) | 20.9(10) | 19.3(10) | -9.8(8) | 0.4(8) | -5.7(8) |
| C34 | 24.9(10) | 24.8(10) | 28.9(11) | -13.7(9) | -2.2(9) | -2.5(8) |

**Table S5.** Bond Lengths in Å for **MLX III**.

| Atom | Atom | Length [Å] | Atom | Atom | Length [Å] |
|---|---|---|---|---|---|
| S1 | O1 | 1.4322(15) | S21 | O21 | 1.4335(15) |
| S1 | O2 | 1.4307(15) | S21 | O22 | 1.4306(15) |
| S1 | N1 | 1.6445(17) | S21 | N21 | 1.6462(17) |
| S1 | C1 | 1.760(2) | S21 | C21 | 1.755(2) |
| S2 | C11 | 1.7383(19) | S22 | C31 | 1.738(2) |
| S2 | C13 | 1.730(2) | S22 | C33 | 1.732(2) |
| O3 | C10 | 1.254(2) | O23 | C30 | 1.249(2) |
| O7 | C7 | 1.342(2) | O27 | C27 | 1.340(2) |
| N1 | C8 | 1.440(2) | N21 | C28 | 1.440(2) |
| N1 | C9 | 1.486(3) | N21 | C29 | 1.486(3) |
| N2 | C10 | 1.361(3) | N22 | C30 | 1.358(3) |
| N2 | C11 | 1.391(3) | N22 | C31 | 1.392(3) |
| N3 | C11 | 1.304(3) | N23 | C31 | 1.308(3) |
| N3 | C12 | 1.387(3) | N23 | C32 | 1.384(3) |
| C1 | C2 | 1.383(3) | C21 | C22 | 1.384(3) |
| C1 | C6 | 1.403(3) | C21 | C26 | 1.406(3) |
| C2 | C3 | 1.392(3) | C22 | C23 | 1.390(3) |
| C3 | C4 | 1.391(3) | C23 | C24 | 1.394(3) |
| C4 | C5 | 1.385(3) | C24 | C25 | 1.384(3) |
| C5 | C6 | 1.399(3) | C25 | C26 | 1.396(3) |
| C6 | C7 | 1.462(3) | C26 | C27 | 1.465(3) |
| C7 | C8 | 1.368(3) | C27 | C28 | 1.370(3) |
| C8 | C10 | 1.455(3) | C28 | C30 | 1.459(3) |
| C12 | C13 | 1.352(3) | C32 | C33 | 1.351(3) |

| Atom | Atom | Length [Å] | Atom | Atom | Length [Å] |
|---|---|---|---|---|---|
| C13 | C14 | 1.497(3) | C33 | C34 | 1.501(3) |

**Table S6.** Bond Angles in ° for **MLX III**.

| Atom | Atom | Atom | Angle [°] | Atom | Atom | Atom | Angle [°] |
|---|---|---|---|---|---|---|---|
| O1 | S1 | N1 | 106.56(8) | O21 | S21 | N21 | 107.00(9) |
| O1 | S1 | C1 | 107.45(9) | O21 | S21 | C21 | 107.47(9) |
| O2 | S1 | O1 | 119.61(9) | O22 | S21 | O21 | 119.44(9) |
| O2 | S1 | N1 | 108.69(9) | O22 | S21 | N21 | 108.48(9) |
| O2 | S1 | C1 | 110.12(9) | O22 | S21 | C21 | 109.96(9) |
| N1 | S1 | C1 | 103.12(9) | N21 | S21 | C21 | 103.26(9) |
| C13 | S2 | C11 | 89.18(9) | C33 | S22 | C31 | 89.12(10) |
| C8 | N1 | S1 | 113.34(13) | C28 | N21 | S21 | 112.39(13) |
| C8 | N1 | C9 | 115.63(15) | C28 | N21 | C29 | 114.44(16) |
| C9 | N1 | S1 | 116.83(13) | C29 | N21 | S21 | 116.94(14) |
| C10 | N2 | C11 | 122.22(16) | C30 | N22 | C31 | 122.42(16) |
| C11 | N3 | C12 | 109.55(16) | C31 | N23 | C32 | 109.15(17) |
| C2 | C1 | S1 | 121.10(15) | C22 | C21 | S21 | 120.88(15) |
| C2 | C1 | C6 | 122.25(18) | C22 | C21 | C26 | 122.36(18) |
| C6 | C1 | S1 | 116.42(15) | C26 | C21 | S21 | 116.46(15) |
| C1 | C2 | C3 | 118.93(19) | C21 | C22 | C23 | 118.82(18) |
| C4 | C3 | C2 | 119.88(19) | C22 | C23 | C24 | 119.70(19) |
| C5 | C4 | C3 | 120.77(19) | C25 | C24 | C23 | 121.09(19) |
| C4 | C5 | C6 | 120.44(19) | C24 | C25 | C26 | 120.25(18) |
| C1 | C6 | C7 | 120.79(18) | C21 | C26 | C27 | 120.58(17) |
| C5 | C6 | C1 | 117.73(18) | C25 | C26 | C21 | 117.75(18) |
| C5 | C6 | C7 | 121.48(18) | C25 | C26 | C27 | 121.63(17) |
| O7 | C7 | C6 | 114.00(17) | O27 | C27 | C26 | 114.36(17) |
| O7 | C7 | C8 | 122.76(18) | O27 | C27 | C28 | 122.47(18) |
| C8 | C7 | C6 | 123.25(18) | C28 | C27 | C26 | 123.15(17) |

| Atom | Atom | Atom | Angle [˚] | Atom | Atom | Atom | Angle [˚] |
|---|---|---|---|---|---|---|---|
| N1 | C8 | C10 | 118.47(17) | N21 | C28 | C30 | 118.88(17) |
| C7 | C8 | N1 | 121.44(18) | C27 | C28 | N21 | 121.07(17) |
| C7 | C8 | C10 | 120.09(17) | C27 | C28 | C30 | 120.05(17) |
| O3 | C10 | N2 | 120.24(18) | O23 | C30 | N22 | 120.57(18) |
| O3 | C10 | C8 | 120.78(18) | O23 | C30 | C28 | 120.28(18) |
| N2 | C10 | C8 | 118.98(17) | N22 | C30 | C28 | 119.14(17) |
| N2 | C11 | S2 | 123.53(14) | N22 | C31 | S22 | 123.77(15) |
| N3 | C11 | S2 | 115.18(15) | N23 | C31 | S22 | 115.36(15) |
| N3 | C11 | N2 | 121.25(17) | N23 | C31 | N22 | 120.87(17) |
| C13 | C12 | N3 | 116.89(18) | C33 | C32 | N23 | 117.36(18) |
| C12 | C13 | S2 | 109.19(15) | C32 | C33 | S22 | 109.01(15) |
| C12 | C13 | C14 | 128.85(19) | C32 | C33 | C34 | 128.59(19) |
| C14 | C13 | S2 | 121.94(15) | C34 | C33 | S22 | 122.35(15) |

**Table S7**. Torsion Angles in ° for **MLX III**.

| A | B | C | D | Angle [°] | A | B | C | D | Angle [°] |
|---|---|---|---|---|---|---|---|---|---|
| S1 | N1 | C8 | C7 | 38.2(2) | S21 | N21 | C28 | C27 | 41.4(2) |
| S1 | N1 | C8 | C10 | -142.11(15) | S21 | N21 | C28 | C30 | -139.19(16) |
| S1 | C1 | C2 | C3 | -172.95(16) | S21 | C21 | C22 | C23 | -171.56(16) |
| S1 | C1 | C6 | C5 | 173.39(15) | S21 | C21 | C26 | C25 | 173.31(15) |
| S1 | C1 | C6 | C7 | -6.8(3) | S21 | C21 | C26 | C27 | -4.4(3) |
| O1 | S1 | N1 | C8 | 61.79(15) | O21 | S21 | N21 | C28 | 60.41(15) |
| O1 | S1 | N1 | C9 | -159.94(14) | O21 | S21 | N21 | C29 | -164.24(14) |
| O1 | S1 | C1 | C2 | 99.09(18) | O21 | S21 | C21 | C22 | 96.87(18) |
| O1 | S1 | C1 | C6 | -75.44(17) | O21 | S21 | C21 | C26 | -76.93(17) |
| O2 | S1 | N1 | C8 | -168.06(13) | O22 | S21 | N21 | C28 | -169.48(13) |
| O2 | S1 | N1 | C9 | -29.79(17) | O22 | S21 | N21 | C29 | -34.14(17) |
| O2 | S1 | C1 | C2 | -32.7(2) | O22 | S21 | C21 | C22 | -34.6(2) |
| O2 | S1 | C1 | C6 | 152.73(15) | O22 | S21 | C21 | C26 | 151.57(15) |
| O7 | C7 | C8 | N1 | 179.11(17) | O27 | C27 | C28 | N21 | 178.34(17) |
| O7 | C7 | C8 | C10 | -0.5(3) | O27 | C27 | C28 | C30 | -1.1(3) |
| N1 | S1 | C1 | C2 | -148.58(17) | N21 | S21 | C21 | C22 | -150.23(17) |
| N1 | S1 | C1 | C6 | 36.89(17) | N21 | S21 | C21 | C26 | 35.97(17) |
| N1 | C8 | C10 | O3 | -172.99(17) | N21 | C28 | C30 | O23 | -175.65(17) |
| N1 | C8 | C10 | N2 | 6.5(3) | N21 | C28 | C30 | N22 | 3.4(3) |
| N3 | C12 | C13 | S2 | -0.5(2) | N23 | C32 | C33 | S22 | -0.3(2) |
| N3 | C12 | C13 | C14 | 177.63(19) | N23 | C32 | C33 | C34 | 177.07(19) |
| | | | | | | | | | |
| C1 | S1 | N1 | C8 | -51.20(15) | C21 | S21 | N21 | C28 | -52.83(15) |
| C1 | S1 | N1 | C9 | 87.07(15) | C21 | S21 | N21 | C29 | 82.52(15) |
| C1 | C2 | C3 | C4 | -0.5(3) | C21 | C22 | C23 | C24 | -2.1(3) |
| C1 | C6 | C7 | O7 | 164.47(18) | C21 | C26 | C27 | O27 | 162.21(17) |
| C1 | C6 | C7 | C8 | -15.0(3) | C21 | C26 | C27 | C28 | -15.9(3) |
| C2 | C1 | C6 | C5 | -1.1(3) | C22 | C21 | C26 | C25 | -0.4(3) |
| C2 | C1 | C6 | C7 | 178.78(19) | C22 | C21 | C26 | C27 | -178.14(19) |

| A | B | C | D | Angle [°] | A | B | C | D | Angle [°] |
|---|---|---|---|---|---|---|---|---|---|
| C2 | C3 | C4 | C5 | -0.4(3) | C22 | C23 | C24 | C25 | 0.8(3) |
| C3 | C4 | C5 | C6 | 0.6(3) | C23 | C24 | C25 | C26 | 0.7(3) |
| C4 | C5 | C6 | C1 | 0.1(3) | C24 | C25 | C26 | C21 | -0.9(3) |
| C4 | C5 | C6 | C7 | -179.73(19) | C24 | C25 | C26 | C27 | 176.84(18) |
| C5 | C6 | C7 | O7 | -15.7(3) | C25 | C26 | C27 | O27 | -15.5(3) |
| C5 | C6 | C7 | C8 | 164.9(2) | C25 | C26 | C27 | C28 | 166.44(19) |
| C6 | C1 | C2 | C3 | 1.3(3) | C26 | C21 | C22 | C23 | 1.9(3) |
| C6 | C7 | C8 | N1 | -1.5(3) | C26 | C27 | C28 | N21 | -3.7(3) |
| C6 | C7 | C8 | C10 | 178.86(18) | C26 | C27 | C28 | C30 | 176.85(18) |
| C7 | C8 | C10 | O3 | 6.7(3) | C27 | C28 | C30 | O23 | 3.8(3) |
| C7 | C8 | C10 | N2 | -173.81(18) | C27 | C28 | C30 | N22 | -177.12(18) |
| C9 | N1 | C8 | C7 | -100.6(2) | C29 | N21 | C28 | C27 | -95.2(2) |
| C9 | N1 | C8 | C10 | 79.1(2) | C29 | N21 | C28 | C30 | 84.3(2) |
| C10 | N2 | C11 | S2 | -2.6(3) | C30 | N22 | C31 | S22 | -7.9(3) |
| C10 | N2 | C11 | N3 | -179.92(18) | C30 | N22 | C31 | N23 | 172.98(18) |
| C11 | S2 | C13 | C12 | -0.03(15) | C31 | S22 | C33 | C32 | 0.20(16) |
| C11 | S2 | C13 | C14 | -178.32(17) | C31 | S22 | C33 | C34 | -177.38(18) |
| C11 | N2 | C10 | O3 | -2.1(3) | C31 | N22 | C30 | O23 | 4.2(3) |
| C11 | N2 | C10 | C8 | 178.35(17) | C31 | N22 | C30 | C28 | -174.88(17) |
| C11 | N3 | C12 | C13 | 1.0(3) | C31 | N23 | C32 | C33 | 0.3(3) |
| C12 | N3 | C11 | S2 | -1.0(2) | C32 | N23 | C31 | S22 | -0.1(2) |
| C12 | N3 | C11 | N2 | 176.55(17) | C32 | N23 | C31 | N22 | 179.12(17) |
| C13 | S2 | C11 | N2 | -176.85(17) | C33 | S22 | C31 | N22 | -179.25(18) |
| C13 | S2 | C11 | N3 | 0.60(16) | C33 | S22 | C31 | N23 | -0.06(16) |

**Table S8**. Hydrogen Fractional Atomic Coordinates (×10[4]) and Equivalent Isotropic Displacement Parameters (Å$^2$×10$^3$) for **MLX III**. $U_{eq}$ is defined as 1/3 of the trace of the orthogonalized $U_{ij}$.

| Atom | x | y | z | $U_{eq}$ |
|---|---|---|---|---|
| H7 | 10184.89 | 7273.93 | 4525.4 | 38 |
| H2 | 7778.27 | 5545.78 | 3354.36 | 23 |
| H2A | 5046.48 | 8829.05 | 6172.32 | 26 |
| H3 | 5941.7 | 9580.81 | 7300.72 | 28 |
| H4 | 8044.2 | 9382.71 | 7373.36 | 31 |
| H5 | 9268.01 | 8425.31 | 6352.57 | 28 |
| H9A | 7011.54 | 5123.28 | 6180.35 | 38 |
| H9B | 7369.12 | 4512.89 | 5295.59 | 38 |
| H9C | 5985.24 | 5100.72 | 5404.45 | 38 |
| H12 | 9466.43 | 3742.59 | 1011.99 | 24 |
| H14A | 12144.3 | 4926.9 | 456.19 | 35 |
| H14B | 11705.1 | 3628.72 | 514.53 | 35 |
| H14C | 12524.65 | 3554.03 | 1489.57 | 35 |
| | | | | |
| H27 | 4697.63 | 2282.97 | 832.48 | 33 |
| H22 | 7046.24 | 3954.15 | 2110.43 | 24 |
| H22A | 9879.3 | -1100 | 1107.93 | 26 |
| H23 | 9028.17 | -2599.31 | 684.37 | 26 |
| H24 | 6924.54 | -2355.68 | 546.62 | 26 |
| H25 | 5659.68 | -613.37 | 775.09 | 25 |
| H29A | 7715.09 | 229.67 | 3390.56 | 41 |
| H29B | 7449.83 | 1578.56 | 3558.77 | 41 |
| H29C | 8811.05 | 933.39 | 3439.24 | 41 |
| H32 | 5194.65 | 7470.93 | 2810.14 | 25 |
| H34A | 2564.12 | 8462.2 | 1213.21 | 38 |
| H34B | 2978.94 | 8565.72 | 2296.16 | 38 |
| H34C | 2194 | 7478.51 | 2387.35 | 38 |

**Table S9.** Hydrogen Bond information for **MLX III**.

| D | H | A | d(D-H) [Å] | d(H-A) [Å] | d(D-A) [Å] | D-H-A [°] |
|---|---|---|---|---|---|---|
| O7 | H7 | O3 | 0.84 | 1.80 | 2.536(2) | 145.9 |
| O27 | H27 | O23 | 0.84 | 1.78 | 2.517(2) | 146.0 |

## CSP attempts to reproduce MLX-III

### *Rigid CSP searches*

Table S10 summarizes the results of the CSP searches performed in *P*-1 space group in the search for MLX-III structure using different combination of three conformations of MLX: gas phase minimum conformation (mMIN) and two conformations taken directly from the experimentally determined crystal structure corresponding to two symmetry independent molecules A and B (mA and mB, respectively). The differences between these three considered conformations are indeed very small, which is graphically represented in their molecular overlays shown in Figure S7. The RMSD values in terms of atomic positions representing the differences between mA, mB and mMIN are given in Table S10. Also, Table S11 lists torsion angle values for mA, mB and mMIN. Relative lattice energies given in Table S10 are taken from the force field values for the structure most closely resembling the experimental crystal structure of MLX-II generated in a given attempt, and in respect to the force field global minimum structure, which is the structure of MLX-I. In each case we compared all lowest energy structures with relative lattice energy within 20 kJ/mol from the lowest energy structure of a given attempt. The cluster size of the best match structure and the RMSD value of the best match are from Crystal Packing Similarity Tool [2].

**Table S10.** Summary of the results of rigid CSP attempts to generate MLX-III structure using slightly different conformations of MLX. Bold font marks the only attempt in which the experimental crystal structure of MLX-III was found.

| conformations* | $RMSD_1$ (Å) difference in respect to the scXRD structure | relative lattice energy (kJ/mol)** | cluster size of the best match | RMSD of the best match (Å) | experimental structure found? |
|---|---|---|---|---|---|
| **mA_mB** | **0\|0** | **4.68** | **15/15** | **0.331** | **Yes** |
| mA_mA | 0\|0.09 | 12.88 | 11/15 | 0.468 | No |
| mB_mB | 0.09\|0 | 22.60 | 6/15 | 0.433 | No |
| mA_mMIN | 0\|0.177 | 17.50 | 4/15 | 0.539 | No |
| mB_mMIN | 0.188\|0 | 19.04 | 4/15 | 1.033 | No |
| mMIN_mMIN | 0.177\|0.188 | 19.45 | 7/15 | 0.570 | No |

* mA and mB are experimental conformations extracted from form III; mMIN is gas phase minimum conformation, present also in the experimental form I of meloxicam (MLX)

** in respect to the force field global minimum (GM)

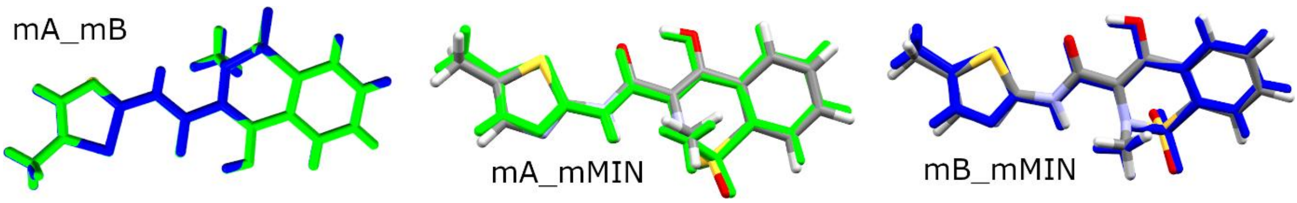


**Figure S7.** Molecular overlays of conformations mA, mB and mMIN.

**Table S11.** Comparison of torsion angle values of the considered conformations

| torsion angle | | | | mMIN | mA | mB |
|---|---|---|---|---|---|---|
| O2 | S1 | N1 | C8 | 60.9 | 61.8 | 60.5 |
| O2 | S1 | N1 | C9 | -165.3 | -159.9 | -164.3 |
| C1 | S1 | N1 | C8 | -53.8 | -51.2 | -52.7 |
| C1 | S1 | N1 | C9 | 80.0 | 87.2 | 82.5 |
| O4 | S1 | N1 | C8 | -169.2 | -168.1 | -169.5 |
| O4 | S1 | N1 | C9 | -35.5 | -29.8 | -34.3 |
| N1 | S1 | C1 | C6 | 38.4 | 36.9 | 36.0 |
| N1 | S1 | C1 | C2 | -143.9 | -148.7 | -150.3 |
| O2 | S1 | C1 | C6 | -75.3 | -75.4 | -76.8 |
| O2 | S1 | C1 | C2 | 102.4 | 99.0 | 96.9 |
| O4 | S1 | C1 | C6 | 152.9 | 152.7 | 151.7 |
| O4 | S1 | C1 | C2 | -29.4 | -32.8 | -34.6 |
| C13 | S2 | C11 | N2 | -179.1 | -176.8 | -179.2 |
| C13 | S2 | C11 | N3 | 0.1 | 0.5 | -0.2 |
| C11 | S2 | C13 | C12 | -0.5 | 0.0 | 0.3 |
| C11 | S2 | C13 | C14 | 179.7 | -178.3 | -177.5 |
| S1 | N1 | C8 | C7 | 40.4 | 38.3 | 41.2 |
| S1 | N1 | C8 | C10 | -141.3 | -142.3 | -139.4 |
| C9 | N1 | C8 | C7 | -93.4 | -100.6 | -95.1 |
| C9 | N1 | C8 | C10 | 84.8 | 78.8 | 84.3 |
| C10 | N2 | C11 | S2 | 1.5 | -2.7 | -8.0 |
| C10 | N2 | C11 | N3 | -177.6 | -179.9 | 173.0 |

| | | | | | | |
|---|---|---|---|---|---|---|
| H12 | N2 | C11 | S2 | 173.1 | 177.2 | 172.0 |
| H12 | N2 | C11 | N3 | -6.1 | 0.1 | -7.0 |
| C11 | N2 | C10 | C8 | 177.6 | 178.2 | -174.9 |
| C11 | N2 | C10 | O3 | -2.4 | -1.9 | 4.3 |
| H12 | N2 | C10 | C8 | 6.1 | -1.7 | 5.1 |
| H12 | N2 | C10 | O3 | -174.1 | 178.1 | -175.7 |
| N1 | C8 | C7 | O1 | 178.1 | 179.0 | 178.3 |
| N1 | C8 | C7 | C6 | -1.6 | -1.6 | -3.6 |
| C10 | C8 | C7 | O1 | -0.1 | -0.4 | -1.1 |
| C10 | C8 | C7 | C6 | -179.8 | 179.1 | 177.0 |
| N1 | C8 | C10 | N2 | 6.0 | 6.8 | 3.5 |
| N1 | C8 | C10 | O3 | -174.0 | -173.1 | -175.7 |
| C7 | C8 | C10 | N2 | -175.8 | -173.8 | -177.0 |
| C7 | C8 | C10 | O3 | 4.3 | 6.3 | 3.7 |
| C12 | N3 | C11 | S2 | 0.3 | -0.9 | 0.0 |
| C12 | N3 | C11 | N2 | 179.5 | 176.5 | 179.1 |
| C11 | N3 | C12 | C13 | -0.7 | 0.9 | 0.3 |
| C8 | C7 | O1 | H13 | -3.1 | -3.5 | -3.5 |
| C6 | C7 | O1 | H13 | 177.1 | 177.1 | 178.2 |
| C8 | C7 | C6 | C1 | -16.0 | -15.0 | -15.9 |
| C8 | C7 | C6 | C5 | 162.6 | 165.1 | 166.4 |
| O1 | C7 | C6 | C1 | 164.3 | 164.5 | 162.4 |
| O1 | C7 | C6 | C5 | -17.2 | -15.4 | -15.4 |
| C7 | C6 | C1 | S1 | -6.7 | -6.7 | -4.5 |
| C7 | C6 | C1 | C2 | 175.6 | 178.9 | -178.1 |
| C5 | C6 | C1 | S1 | 174.7 | 173.2 | 173.3 |
| C5 | C6 | C1 | C2 | -3.0 | -1.2 | -0.3 |
| C7 | C6 | C5 | C4 | -177.0 | -179.7 | 176.8 |
| C1 | C6 | C5 | C4 | 1.5 | 0.3 | -1.0 |
| S1 | C1 | C2 | C3 | -175.4 | -172.9 | -171.6 |
| C6 | C1 | C2 | C3 | 2.2 | 1.2 | 1.7 |
| N3 | C12 | C13 | S2 | 0.8 | -0.5 | -0.4 |
| N3 | C12 | C13 | C14 | -179.4 | 177.7 | 177.2 |

| | | | | | | |
|---|---|---|---|---|---|---|
| C1 | C2 | C3 | C4 | 0.1 | -0.4 | -1.9 |
| C6 | C5 | C4 | C3 | 0.6 | 0.5 | 0.8 |
| C5 | C4 | C3 | C2 | -1.4 | -0.4 | 0.7 |

***Flexible CSP results***

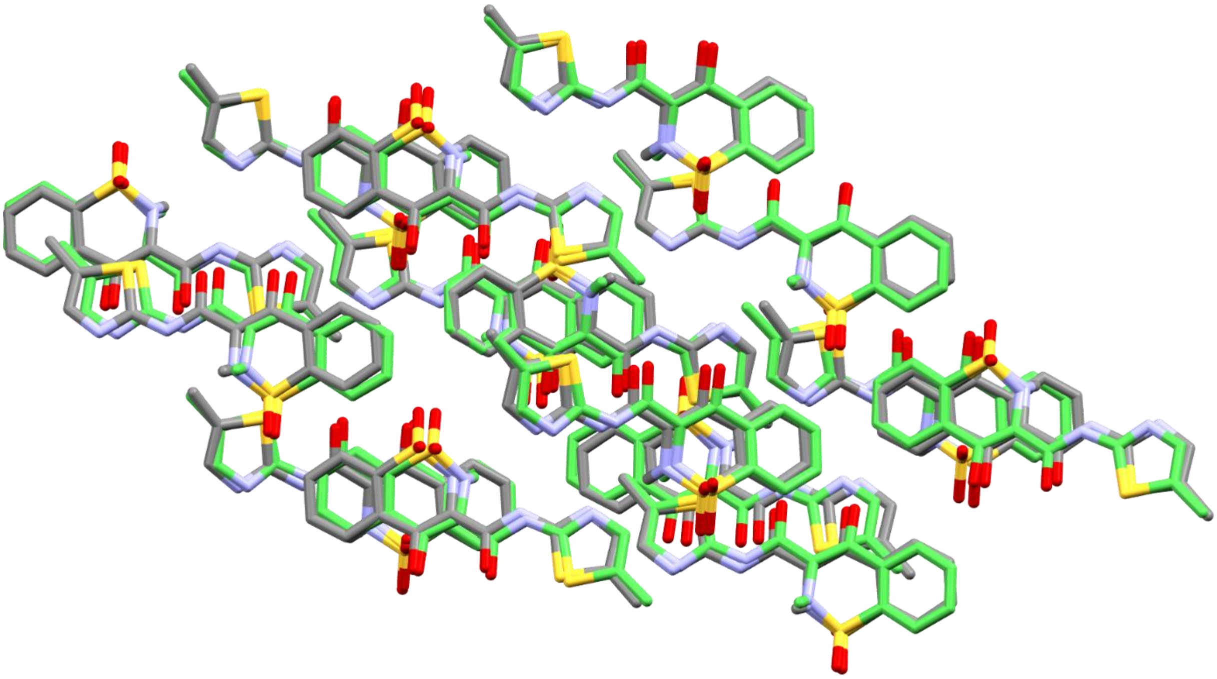

**Figure S8.** An overlay of 15-molecule cluster of MLX-III and a Z' = 2 crystal structer with rank 41 generated with a flexible crystal structure search using CrystalPredictor. The RMSD value for the shown overlay is 0.364 Å.

**Structural data from microED for MLX-V**

**Table S12.** Fractional Atomic Coordinates ($\times10^4$) and Equivalent Isotropic Displacement Parameters ($Å^2\times10^3$) for **MLX-V**. $U_{eq}$ is defined as 1/3 of the trace of the orthogonalised $U_{IJ}$ tensor.

| Atom | *x* | *y* | *z* | U(eq) |
|---|---|---|---|---|
| S62 | 2138(4) | 5193(4) | 9386(3) | 30.6(15) |
| S42 | 3677(4) | 2722(4) | 6610(3) | 30.3(15) |
| S22 | 1324(4) | 3390(4) | 7271(3) | 28.4(14) |
| S2 | 2864(4) | 628(4) | 4799(3) | 33.1(15) |
| S61 | 5648(3) | 1938(3) | 10189(3) | 23.9(13) |

| Atom | *x* | *y* | *z* | U(eq) |
|---|---|---|---|---|
| S41 | 3064(4) | 7423(4) | 5248(3) | 30.5(15) |
| S21 | 1919(4) | 4743(3) | 2571(3) | 24.3(13) |
| S1 | -649(3) | -176(3) | 8055(3) | 22.6(13) |
| O63 | 3765(6) | 4489(7) | 10375(5) | 31(2) |
| O43 | 3711(6) | 4474(7) | 5055(6) | 35(3) |
| O23 | 1269(8) | 4924(8) | 5543(6) | 42(3) |
| O3 | 1229(7) | -379(7) | 5488(5) | 34(3) |
| O67 | 5003(7) | 3651(7) | 11609(5) | 34(2) |
| O47 | 3584(7) | 6071(8) | 3508(6) | 36(2) |
| O27 | 1439(7) | 6507(7) | 3952(6) | 33(2) |
| O7 | -19(8) | -1597(7) | 6366(6) | 40(2) |
| O62 | 6175(7) | 1647(7) | 9550(5) | 38(3) |
| O42 | 3342(8) | 7942(8) | 5753(6) | 45(3) |
| O21 | 2903(6) | 4689(6) | 2808(5) | 31(2) |
| O1 | 169(6) | -678(6) | 8575(5) | 25(2) |
| O61 | 4811(7) | 1422(7) | 10678(6) | 39(3) |
| O41 | 2100(7) | 7168(7) | 5295(5) | 40(3) |
| O22 | 1683(7) | 4264(7) | 2056(6) | 40(3) |
| O2 | -1173(6) | 438(6) | 8344(5) | 29(2) |
| N63 | 2739(7) | 5170(7) | 7823(6) | 31(2) |
| N43 | 3834(7) | 3174(7) | 7961(5) | 27(2) |
| N23 | 1154(7) | 2039(6) | 6816(5) | 24(2) |
| N3 | 2280(7) | 2176(7) | 4839(6) | 29(2) |
| N62 | 3681(7) | 4214(7) | 9102(5) | 30(2) |
| N42 | 3693(7) | 4536(7) | 6476(5) | 29(2) |
| N22 | 1314(7) | 3535(7) | 5468(5) | 33(2) |
| N2 | 1286(7) | 895(8) | 5742(6) | 36(3) |
| N61 | 5313(6) | 3066(6) | 9602(5) | 27(2) |
| N41 | 3731(6) | 6468(6) | 5598(5) | 20(2) |
| N21 | 1289(7) | 4401(7) | 3531(6) | 33(2) |
| N1 | -330(7) | 411(6) | 6917(5) | 25(2) |
| C74 | 569(8) | 6353(9) | 8406(7) | 42(3) |
| C54 | 3654(9) | 786(8) | 7984(8) | 49(3) |
| C34 | 1266(10) | 1968(10) | 9179(7) | 58(4) |
| C14 | 4422(8) | 1545(9) | 3624(8) | 53(3) |
| C73 | 1477(7) | 5823(7) | 8391(6) | 25(2) |
| C53 | 3734(8) | 1809(8) | 7776(7) | 30(3) |
| C33 | 1274(8) | 2245(8) | 8175(6) | 30(3) |
| C13 | 3524(8) | 1607(8) | 4183(7) | 36(3) |
| C72 | 1893(7) | 5699(7) | 7646(6) | 30(3) |

| Atom | *x* | *y* | *z* | U(eq) |
|---|---|---|---|---|
| C52 | 3845(7) | 2237(7) | 8357(7) | 27(3) |
| C32 | 1168(8) | 1655(8) | 7785(6) | 34(3) |
| C12 | 3100(8) | 2373(8) | 4302(6) | 36(3) |
| C71 | 2919(7) | 4826(7) | 8722(6) | 26(3) |
| C51 | 3744(7) | 3543(7) | 7035(6) | 25(3) |
| C31 | 1249(8) | 2953(7) | 6463(6) | 27(3) |
| C11 | 2077(8) | 1239(8) | 5177(7) | 40(3) |
| C70 | 4105(7) | 4091(7) | 9912(6) | 23.3(17) |
| C50 | 3664(7) | 4929(7) | 5533(6) | 23.3(17) |
| C30 | 1304(8) | 4479(8) | 5049(7) | 34(3) |
| C10 | 916(7) | 102(7) | 5895(6) | 21(2) |
| C68 | 4932(7) | 3449(7) | 10213(6) | 24(2) |
| C48 | 3670(7) | 5957(8) | 5057(6) | 26(3) |
| C28 | 1361(8) | 4967(8) | 4028(6) | 29(3) |
| C8 | 90(8) | -220(7) | 6542(6) | 27(3) |
| C67 | 5308(8) | 3265(7) | 11057(6) | 27(3) |
| C47 | 3624(7) | 6459(7) | 4073(5) | 17(2) |
| C27 | 1391(8) | 5919(8) | 3518(7) | 35(3) |
| C7 | -368(7) | -1071(7) | 6746(6) | 26(3) |
| C66 | 6148(7) | 2591(7) | 11420(6) | 22(2) |
| C46 | 3641(7) | 7520(7) | 3557(6) | 24(2) |
| C26 | 1397(7) | 6442(7) | 2497(6) | 22.1(17) |
| C6 | -1164(7) | -1391(7) | 7397(6) | 25(3) |
| C65 | 6688(8) | 2570(8) | 12123(7) | 41(3) |
| C45 | 3823(7) | 8004(7) | 2600(6) | 26(2) |
| C25 | 1159(8) | 7399(7) | 2007(7) | 36(3) |
| C5 | -1668(7) | -2120(7) | 7438(6) | 30(3) |
| C64 | 7485(8) | 1917(7) | 12433(6) | 35(3) |
| C44 | 3906(9) | 8955(9) | 2145(8) | 41(3) |
| C24 | 1111(8) | 7848(8) | 1041(7) | 35(3) |
| C4 | -2484(8) | -2429(8) | 8043(6) | 32(3) |
| C63 | 7751(8) | 1386(9) | 12004(7) | 42(3) |
| C43 | 3773(9) | 9465(9) | 2671(7) | 44(3) |
| C23 | 1207(9) | 7325(8) | 523(7) | 42(3) |
| C3 | -2745(9) | -2006(9) | 8649(7) | 41(3) |
| C62 | 7210(7) | 1390(7) | 11281(6) | 32(3) |
| C42 | 3548(7) | 9018(7) | 3648(6) | 27(3) |
| C22 | 1435(8) | 6378(8) | 974(6) | 40(3) |
| C2 | -2232(8) | -1287(8) | 8631(6) | 32(3) |
| C61 | 6410(7) | 1963(7) | 11006(6) | 27(3) |

| Atom | *x* | *y* | *z* | U(eq) |
|---|---|---|---|---|
| C41 | 3447(8) | 8045(8) | 4075(6) | 32(3) |
| C21 | 1575(7) | 5917(7) | 1965(6) | 22.1(17) |
| C1 | -1399(7) | -994(7) | 8021(6) | 27(2) |
| C69 | 5986(8) | 3670(7) | 8868(7) | 36(3) |
| C49 | 4661(8) | 6471(8) | 5890(7) | 36(3) |
| C29 | 306(8) | 4078(8) | 3504(6) | 38(3) |
| C9 | -941(8) | 1148(7) | 6314(6) | 33(3) |

**Table S13.** Anisotropic Displacement Parameters ($Å^2×10^3$) for **MLX-V**. The Anisotropic displacement factor exponent takes the form: -2π2[h2a*2$U_{11}$+2hka*b*$U_{12}$+…].

| Atom | $U_{11}$ | $U_{22}$ | $U_{33}$ | $U_{12}$ | $U_{13}$ | $U_{23}$ |
|---|---|---|---|---|---|---|
| S62 | 36(3) | 36(3) | 20(2) | 12(2) | -6.4(18) | -14.1(19) |
| S42 | 30(3) | 28(3) | 32(2) | 6(2) | 1.2(19) | -13(2) |
| S22 | 24(3) | 39(3) | 22(2) | -1(2) | 1.3(18) | -14(2) |
| S2 | 38(3) | 26(3) | 34(3) | -5(2) | 9(2) | -14(2) |
| S61 | 21(3) | 24(3) | 17(2) | 5.9(19) | -1.7(17) | -1.3(17) |
| S41 | 28(3) | 34(3) | 22(2) | 10(2) | 4.4(18) | -8.6(19) |
| S21 | 32(3) | 19(3) | 25(2) | 0(2) | 0.5(19) | -14.3(19) |
| S1 | 21(3) | 21(3) | 20(2) | -1.0(19) | 3.2(17) | -4.8(17) |
| O63 | 29(5) | 37(6) | 26(4) | 2(4) | -7(3) | -13(3) |
| O43 | 21(5) | 41(6) | 32(4) | 1(4) | -6(3) | -6(4) |
| O23 | 52(7) | 48(6) | 23(4) | 3(4) | -1(4) | -14(4) |
| O3 | 35(5) | 45(6) | 18(4) | 3(4) | 14(3) | -12(3) |
| O67 | 43(5) | 34(5) | 18(3) | 7(3) | 5(3) | -9(3) |
| O47 | 33(5) | 43(6) | 30(4) | -7(3) | -1(3) | -14(3) |
| O27 | 44(5) | 34(5) | 29(4) | 11(3) | -7(3) | -23(3) |
| O7 | 41(5) | 31(5) | 34(4) | 3(3) | 3(3) | -5(3) |
| O62 | 44(6) | 32(5) | 32(4) | 21(4) | -6(3) | -12(3) |
| O42 | 51(7) | 57(7) | 28(4) | 18(5) | 0(4) | -23(4) |
| O21 | 22(5) | 41(6) | 22(4) | 4(4) | 9(3) | -10(3) |
| O1 | 14(5) | 37(5) | 21(4) | 1(3) | -6(3) | -9(3) |
| O61 | 31(6) | 38(6) | 36(5) | 3(4) | -14(4) | -4(4) |
| O41 | 42(6) | 45(6) | 23(4) | 14(4) | -3(3) | -8(4) |
| O22 | 33(5) | 41(6) | 28(4) | 6(4) | 14(3) | -3(4) |
| O2 | 28(5) | 32(5) | 17(4) | 7(4) | 2(3) | -3(3) |
| N63 | 23(5) | 40(5) | 31(4) | 3(3) | -1(3) | -18(3) |
| N43 | 20(5) | 29(5) | 23(4) | -3(3) | -5(3) | -3(3) |
| N23 | 36(5) | 22(5) | 13(3) | -6(3) | 5(3) | -7(3) |
| N3 | 16(5) | 38(5) | 36(4) | -6(3) | 5(3) | -20(3) |
| N62 | 26(5) | 37(5) | 29(4) | 14(3) | -4(3) | -20(3) |

| Atom | $U_{11}$ | $U_{22}$ | $U_{33}$ | $U_{12}$ | $U_{13}$ | $U_{23}$ |
|---|---|---|---|---|---|---|
| N42 | 40(5) | 27(5) | 21(4) | 5(3) | -3(3) | -12(3) |
| N22 | 50(6) | 40(6) | 14(3) | 0(3) | 1(3) | -17(3) |
| N2 | 29(5) | 41(6) | 33(4) | 3(3) | 5(3) | -13(3) |
| N61 | 18(4) | 25(5) | 25(4) | 0(3) | 5(3) | -1(3) |
| N41 | 17(4) | 22(5) | 19(3) | -1(3) | 3(3) | -8(3) |
| N21 | 39(5) | 36(5) | 27(4) | 5(3) | 7(3) | -19(3) |
| N1 | 28(5) | 23(5) | 20(4) | 4(3) | -2(3) | -5(3) |
| C74 | 36(6) | 48(6) | 44(5) | 6(4) | -9(3) | -23(3) |
| C54 | 39(6) | 47(6) | 57(6) | 8(4) | -10(4) | -19(3) |
| C34 | 53(7) | 65(7) | 54(6) | 4(4) | 0(4) | -26(4) |
| C14 | 45(6) | 56(7) | 62(6) | -2(4) | 11(4) | -31(4) |
| C73 | 21(5) | 38(6) | 21(4) | 2(3) | 0(3) | -19(3) |
| C53 | 24(5) | 25(6) | 41(5) | -3(3) | -4(3) | -14(3) |
| C33 | 34(6) | 40(6) | 22(4) | 3(4) | 4(3) | -20(3) |
| C13 | 28(6) | 36(6) | 42(5) | -8(3) | 11(3) | -17(3) |
| C72 | 30(5) | 23(5) | 23(4) | 17(3) | -9(3) | 0(3) |
| C52 | 24(5) | 24(5) | 26(4) | 4(3) | -2(3) | -7(3) |
| C32 | 39(6) | 27(6) | 24(4) | 3(3) | 2(3) | -3(3) |
| C12 | 32(6) | 34(6) | 38(5) | -3(4) | 4(3) | -13(3) |
| C71 | 32(6) | 29(5) | 15(4) | -1(3) | 0(3) | -8(3) |
| C51 | 22(5) | 32(6) | 21(4) | 7(3) | -1(3) | -12(3) |
| C31 | 33(6) | 20(5) | 16(4) | 1(3) | -7(3) | 4(3) |
| C11 | 36(6) | 39(6) | 37(5) | -2(4) | 3(3) | -11(3) |
| C70 | 29(4) | 28(4) | 19(3) | 2(2) | 5(2) | -16(2) |
| C50 | 29(4) | 28(4) | 19(3) | 2(2) | 5(2) | -16(2) |
| C30 | 49(6) | 28(6) | 24(4) | -10(4) | 6(3) | -9(3) |
| C10 | 25(5) | 15(5) | 16(4) | 5(3) | 0(3) | -2(3) |
| C68 | 15(5) | 35(6) | 24(4) | 2(3) | 3(3) | -16(3) |
| C48 | 22(5) | 37(6) | 23(4) | -3(3) | 3(3) | -16(3) |
| C28 | 29(6) | 25(5) | 30(4) | 5(3) | -8(3) | -10(3) |
| C8 | 33(5) | 22(5) | 16(4) | 2(3) | 4(3) | 1(3) |
| C67 | 32(6) | 27(5) | 14(4) | -1(3) | 1(3) | -2(3) |
| C47 | 18(5) | 22(5) | 12(4) | -2(3) | 2(3) | -8(3) |
| C27 | 49(6) | 18(5) | 29(5) | 0(3) | -9(3) | -2(3) |
| C7 | 23(5) | 19(5) | 27(4) | -3(3) | 5(3) | -3(3) |
| C66 | 22(5) | 23(5) | 17(4) | 1(3) | -4(3) | -5(3) |
| C46 | 19(5) | 25(5) | 31(4) | 3(3) | 2(3) | -16(3) |
| C26 | 19(4) | 24(4) | 21(3) | 0(2) | 7(2) | -10(2) |
| C6 | 11(5) | 23(5) | 34(4) | 3(3) | 2(3) | -7(3) |
| C65 | 43(6) | 47(6) | 38(5) | 13(4) | -11(3) | -24(3) |

| Atom | $U_{11}$ | $U_{22}$ | $U_{33}$ | $U_{12}$ | $U_{13}$ | $U_{23}$ |
|---|---|---|---|---|---|---|
| C45 | 27(5) | 38(6) | 16(4) | 1(3) | -1(3) | -16(3) |
| C25 | 41(6) | 24(6) | 37(5) | 4(3) | -1(3) | -9(3) |
| C5 | 22(5) | 26(5) | 45(5) | 8(3) | 7(3) | -23(3) |
| C64 | 49(6) | 29(6) | 19(4) | 8(3) | -7(3) | -6(3) |
| C44 | 56(7) | 40(6) | 33(5) | -4(4) | 1(4) | -22(3) |
| C24 | 39(6) | 25(6) | 34(5) | 5(3) | -5(3) | -9(3) |
| C4 | 31(5) | 36(6) | 32(4) | -9(3) | 17(3) | -19(3) |
| C63 | 27(6) | 57(7) | 43(5) | 4(4) | 1(3) | -25(3) |
| C43 | 52(6) | 41(6) | 29(5) | 4(4) | 2(3) | -10(3) |
| C23 | 54(6) | 36(6) | 28(5) | 7(4) | -4(3) | -8(3) |
| C3 | 32(6) | 44(6) | 45(5) | -1(4) | 13(3) | -19(3) |
| C62 | 28(5) | 32(6) | 29(4) | 7(3) | 5(3) | -9(3) |
| C42 | 40(6) | 21(5) | 19(4) | 2(3) | -1(3) | -8(3) |
| C22 | 49(6) | 38(6) | 28(4) | -4(4) | 10(3) | -12(3) |
| C2 | 20(5) | 36(6) | 34(5) | 3(3) | 0(3) | -12(3) |
| C61 | 26(5) | 22(5) | 21(4) | 5(3) | -4(3) | 0(3) |
| C41 | 47(6) | 27(6) | 20(4) | 2(4) | -5(3) | -8(3) |
| C21 | 19(4) | 24(4) | 21(3) | 0(2) | 7(2) | -10(2) |
| C1 | 28(5) | 25(5) | 22(4) | 11(3) | 3(3) | -9(3) |
| C69 | 30(6) | 39(6) | 31(4) | 1(3) | 1(3) | -9(3) |
| C49 | 33(6) | 42(6) | 33(5) | 9(4) | -7(3) | -19(3) |
| C29 | 49(6) | 39(6) | 26(4) | 6(4) | 0(3) | -15(3) |
| C9 | 30(6) | 33(5) | 34(4) | 10(3) | 0(3) | -16(3) |

**Table S14.** Bond Lengths for **MLX-V**.

| Atom | Atom | Length/Å | Atom | Atom | Length/Å |
|---|---|---|---|---|---|
| S62 | C73 | 1.700(10) | N41 | C48 | 1.397(14) |
| S62 | C71 | 1.677(9) | N41 | C49 | 1.393(14) |
| S42 | C53 | 1.732(10) | N21 | C28 | 1.414(15) |
| S42 | C51 | 1.683(12) | N21 | C29 | 1.492(16) |
| S22 | C33 | 1.704(11) | N1 | C8 | 1.416(12) |
| S22 | C31 | 1.680(12) | N1 | C9 | 1.380(11) |
| S2 | C13 | 1.683(13) | C74 | C73 | 1.449(11) |
| S2 | C11 | 1.633(11) | C54 | C53 | 1.481(16) |
| S61 | O62 | 1.399(8) | C34 | C33 | 1.417(13) |
| S61 | O61 | 1.422(11) | C14 | C13 | 1.481(12) |
| S61 | N61 | 1.618(9) | C73 | C72 | 1.336(11) |
| S61 | C61 | 1.718(12) | C53 | C52 | 1.358(16) |
| S41 | O42 | 1.435(13) | C33 | C32 | 1.323(16) |

| Atom | Atom | Length/Å | Atom | Atom | Length/Å |
|---|---|---|---|---|---|
| S41 | O41 | 1.404(12) | C13 | C12 | 1.363(13) |
| S41 | N41 | 1.575(8) | C70 | C68 | 1.414(11) |
| S41 | C41 | 1.699(10) | C50 | C48 | 1.426(14) |
| S21 | O21 | 1.419(10) | C30 | C28 | 1.416(12) |
| S21 | O22 | 1.379(13) | C10 | C8 | 1.423(13) |
| S21 | N21 | 1.558(9) | C68 | C67 | 1.343(14) |
| S21 | C21 | 1.681(9) | C48 | C47 | 1.373(11) |
| S1 | O1 | 1.403(8) | C28 | C27 | 1.330(14) |
| S1 | O2 | 1.362(8) | C8 | C7 | 1.401(16) |
| S1 | N1 | 1.627(8) | C67 | C66 | 1.462(12) |
| S1 | C1 | 1.718(13) | C47 | C46 | 1.475(13) |
| O63 | C70 | 1.190(11) | C27 | C26 | 1.419(12) |
| O43 | C50 | 1.226(14) | C7 | C6 | 1.394(12) |
| O23 | C30 | 1.235(15) | C66 | C65 | 1.352(16) |
| O3 | C10 | 1.214(12) | C66 | C61 | 1.401(14) |
| O67 | C67 | 1.278(12) | C46 | C45 | 1.344(11) |
| O47 | C47 | 1.270(14) | C46 | C41 | 1.378(14) |
| O27 | C27 | 1.360(15) | C26 | C25 | 1.356(12) |
| O7 | C7 | 1.254(13) | C26 | C21 | 1.394(13) |
| N63 | C72 | 1.345(11) | C6 | C5 | 1.348(16) |
| N63 | C71 | 1.286(12) | C6 | C1 | 1.369(13) |
| N43 | C52 | 1.299(13) | C65 | C64 | 1.392(12) |
| N43 | C51 | 1.299(11) | C45 | C44 | 1.331(15) |
| N23 | C32 | 1.346(11) | C25 | C24 | 1.345(12) |
| N23 | C31 | 1.285(14) | C5 | C4 | 1.380(13) |
| N3 | C12 | 1.325(12) | C64 | C63 | 1.288(14) |
| N3 | C11 | 1.350(15) | C44 | C43 | 1.362(15) |
| N62 | C71 | 1.338(11) | C24 | C23 | 1.366(15) |
| N62 | C70 | 1.353(13) | C4 | C3 | 1.379(14) |
| N42 | C51 | 1.388(13) | C63 | C62 | 1.390(17) |
| N42 | C50 | 1.312(11) | C43 | C42 | 1.378(11) |
| N22 | C31 | 1.397(11) | C23 | C22 | 1.337(14) |
| N22 | C30 | 1.309(14) | C3 | C2 | 1.356(17) |
| N2 | C11 | 1.333(14) | C62 | C61 | 1.331(12) |
| N2 | C10 | 1.283(15) | C42 | C41 | 1.363(15) |
| N61 | C68 | 1.380(12) | C22 | C21 | 1.397(12) |
| N61 | C69 | 1.436(12) | C2 | C1 | 1.400(13) |

**Table S15.** Bond Angles for **MLX-V**.

| Atom | Atom | Atom | Angle/° | Atom | Atom | Atom | Angle/° |
|---|---|---|---|---|---|---|---|
| C71 | S62 | C73 | 88.8(4) | N62 | C70 | O63 | 120.4(8) |
| C51 | S42 | C53 | 89.6(5) | C68 | C70 | O63 | 121.2(9) |
| C31 | S22 | C33 | 89.3(6) | C68 | C70 | N62 | 118.4(8) |
| C11 | S2 | C13 | 91.3(6) | N42 | C50 | O43 | 124.4(10) |
| O61 | S61 | O62 | 117.2(8) | C48 | C50 | O43 | 119.8(8) |
| N61 | S61 | O62 | 108.4(5) | C48 | C50 | N42 | 115.5(10) |
| N61 | S61 | O61 | 108.0(5) | N22 | C30 | O23 | 120.0(10) |
| C61 | S61 | O62 | 110.0(6) | C28 | C30 | O23 | 121.5(11) |
| C61 | S61 | O61 | 110.1(5) | C28 | C30 | N22 | 118.5(10) |
| C61 | S61 | N61 | 101.9(6) | N2 | C10 | O3 | 122.4(10) |
| O41 | S41 | O42 | 121.4(6) | C8 | C10 | O3 | 118.0(11) |
| N41 | S41 | O42 | 106.8(6) | C8 | C10 | N2 | 119.6(9) |
| N41 | S41 | O41 | 107.5(6) | C70 | C68 | N61 | 117.2(8) |
| C41 | S41 | O42 | 107.2(7) | C67 | C68 | N61 | 124.2(8) |
| C41 | S41 | O41 | 109.5(6) | C67 | C68 | C70 | 118.6(8) |
| C41 | S41 | N41 | 102.7(4) | C50 | C48 | N41 | 119.6(8) |
| O22 | S21 | O21 | 118.3(6) | C47 | C48 | N41 | 119.0(10) |
| N21 | S21 | O21 | 107.3(5) | C47 | C48 | C50 | 121.4(10) |
| N21 | S21 | O22 | 110.1(7) | C30 | C28 | N21 | 116.8(10) |
| C21 | S21 | O21 | 107.6(6) | C27 | C28 | N21 | 118.6(9) |
| C21 | S21 | O22 | 108.9(6) | C27 | C28 | C30 | 124.3(11) |
| C21 | S21 | N21 | 103.6(5) | C10 | C8 | N1 | 116.7(10) |
| O2 | S1 | O1 | 117.8(6) | C7 | C8 | N1 | 119.3(9) |
| N1 | S1 | O1 | 109.5(5) | C7 | C8 | C10 | 123.6(9) |
| N1 | S1 | O2 | 108.9(5) | C68 | C67 | O67 | 125.4(9) |
| C1 | S1 | O1 | 108.8(6) | C66 | C67 | O67 | 113.3(9) |
| C1 | S1 | O2 | 109.8(6) | C66 | C67 | C68 | 121.3(9) |
| C1 | S1 | N1 | 100.8(5) | C48 | C47 | O47 | 124.5(10) |
| C71 | N63 | C72 | 110.2(8) | C46 | C47 | O47 | 112.8(8) |
| C51 | N43 | C52 | 111.5(11) | C46 | C47 | C48 | 122.7(10) |
| C31 | N23 | C32 | 108.9(10) | C28 | C27 | O27 | 121.5(9) |
| C11 | N3 | C12 | 111.5(9) | C26 | C27 | O27 | 112.1(9) |
| C70 | N62 | C71 | 124.3(9) | C26 | C27 | C28 | 126.3(11) |
| C50 | N42 | C51 | 122.1(10) | C8 | C7 | O7 | 118.7(9) |
| C30 | N22 | C31 | 125.3(10) | C6 | C7 | O7 | 118.0(11) |
| C10 | N2 | C11 | 124.0(10) | C6 | C7 | C8 | 123.2(9) |
| C68 | N61 | S61 | 111.8(6) | C65 | C66 | C67 | 122.3(10) |
| C69 | N61 | S61 | 116.6(6) | C61 | C66 | C67 | 118.4(9) |
| C69 | N61 | C68 | 113.9(10) | C61 | C66 | C65 | 119.3(8) |
| C48 | N41 | S41 | 114.0(7) | C45 | C46 | C47 | 123.1(9) |

| Atom | Atom | Atom | Angle/° | Atom | Atom | Atom | Angle/° |
|---|---|---|---|---|---|---|---|
| C49 | N41 | S41 | 118.8(9) | C41 | C46 | C47 | 118.7(8) |
| C49 | N41 | C48 | 114.7(7) | C41 | C46 | C45 | 118.2(9) |
| C28 | N21 | S21 | 113.9(9) | C25 | C26 | C27 | 124.8(9) |
| C29 | N21 | S21 | 115.6(8) | C21 | C26 | C27 | 117.3(9) |
| C29 | N21 | C28 | 117.2(8) | C21 | C26 | C25 | 117.6(8) |
| C8 | N1 | S1 | 111.3(6) | C5 | C6 | C7 | 119.7(10) |
| C9 | N1 | S1 | 119.3(6) | C1 | C6 | C7 | 119.7(12) |
| C9 | N1 | C8 | 115.4(8) | C1 | C6 | C5 | 120.5(9) |
| C74 | C73 | S62 | 123.1(7) | C64 | C65 | C66 | 119.7(11) |
| C72 | C73 | S62 | 109.7(6) | C44 | C45 | C46 | 123.1(10) |
| C72 | C73 | C74 | 127.1(8) | C24 | C25 | C26 | 122.5(11) |
| C54 | C53 | S42 | 121.1(9) | C4 | C5 | C6 | 121.3(10) |
| C52 | C53 | S42 | 106.8(8) | C63 | C64 | C65 | 120.1(11) |
| C52 | C53 | C54 | 132.1(10) | C43 | C44 | C45 | 118.4(10) |
| C34 | C33 | S22 | 126.5(10) | C23 | C24 | C25 | 120.1(11) |
| C32 | C33 | S22 | 108.3(7) | C3 | C4 | C5 | 118.3(12) |
| C32 | C33 | C34 | 125.1(11) | C62 | C63 | C64 | 121.3(10) |
| C14 | C13 | S2 | 120.3(9) | C42 | C43 | C44 | 121.4(12) |
| C12 | C13 | S2 | 109.9(8) | C22 | C23 | C24 | 119.4(10) |
| C12 | C13 | C14 | 129.9(11) | C2 | C3 | C4 | 121.0(10) |
| C73 | C72 | N63 | 115.3(8) | C61 | C62 | C63 | 120.0(10) |
| C53 | C52 | N43 | 117.7(9) | C41 | C42 | C43 | 118.0(10) |
| C33 | C32 | N23 | 117.7(10) | C21 | C22 | C23 | 121.0(11) |
| C13 | C12 | N3 | 113.8(11) | C1 | C2 | C3 | 119.7(10) |
| N63 | C71 | S62 | 115.7(7) | C66 | C61 | S61 | 117.9(7) |
| N62 | C71 | S62 | 122.2(7) | C62 | C61 | S61 | 122.8(9) |
| N62 | C71 | N63 | 122.1(8) | C62 | C61 | C66 | 119.2(10) |
| N43 | C51 | S42 | 114.3(8) | C46 | C41 | S41 | 116.7(8) |
| N42 | C51 | S42 | 124.9(7) | C42 | C41 | S41 | 122.7(8) |
| N42 | C51 | N43 | 120.8(11) | C42 | C41 | C46 | 120.6(9) |
| N23 | C31 | S22 | 115.8(7) | C26 | C21 | S21 | 117.3(7) |
| N22 | C31 | S22 | 122.7(9) | C22 | C21 | S21 | 123.7(8) |
| N22 | C31 | N23 | 121.5(10) | C22 | C21 | C26 | 118.9(9) |
| N3 | C11 | S2 | 113.4(8) | C6 | C1 | S1 | 119.2(8) |
| N2 | C11 | S2 | 125.8(10) | C2 | C1 | S1 | 121.9(9) |
| N2 | C11 | N3 | 120.7(9) | C2 | C1 | C6 | 118.9(12) |

**Table S16.** Hydrogen Bonds for **MLX-V.**

| D | H | A | d(D-H)/Å | d(H-A)/Å | d(D-A)/Å | D-H-A/° |
|---|---|---|---|---|---|---|

| D | H | A | d(D-H)/Å | d(H-A)/Å | d(D-A)/Å | D-H-A/° |
|---|---|---|---|---|---|---|
| O67 | H67 | O63 | 0.8400 | 1.781(17) | 2.487(12) | 140.4(19) |
| O47 | H47 | O43 | 0.8400 | 1.87(2) | 2.571(12) | 140(3) |
| O27 | H27 | O23 | 0.8400 | 1.875(14) | 2.600(13) | 143.8(5) |
| O7 | H7 | O3 | 0.8400 | 1.741(16) | 2.499(15) | 148.9(12) |
| N62 | H62 | N43 | 0.8800 | 2.050(15) | 2.866(14) | 153.8(3) |
| N42 | H42 | N63 | 0.8800 | 2.173(11) | 2.879(12) | 136.8(4) |
| N22 | H22 | N3 | 0.8800 | 2.160(12) | 2.893(13) | 140.5(4) |

**Table S17.** Torsion Angles for **MLX-V**.

| A | B | C | D | Angle/˚ | A | B | C | D | Angle/˚ |
|---|---|---|---|---|---|---|---|---|---|
| S62 | C73 | C72 | N63 | -3.5(11) | O7 | C7 | C6 | C5 | -15.0(11) |
| S62 | C71 | N63 | C72 | -5.1(11) | O7 | C7 | C6 | C1 | 162.0(9) |
| S62 | C71 | N62 | C70 | -23.9(13) | N63 | C72 | C73 | C74 | -179.7(11) |
| S42 | C53 | C52 | N43 | 3.3(8) | N63 | C71 | N62 | C70 | 155.9(13) |
| S42 | C51 | N43 | C52 | -0.5(8) | N43 | C52 | C53 | C54 | -176.0(9) |
| S42 | C51 | N42 | C50 | -5.7(11) | N43 | C51 | N42 | C50 | 175.5(10) |
| S22 | C33 | C32 | N23 | 2.1(8) | N23 | C32 | C33 | C34 | 177.4(10) |
| S22 | C31 | N23 | C32 | -1.8(9) | N23 | C31 | N22 | C30 | 172.1(10) |
| S22 | C31 | N22 | C30 | -9.8(12) | N3 | C12 | C13 | C14 | 178.6(10) |
| S2 | C13 | C12 | N3 | -1.5(10) | N3 | C11 | N2 | C10 | 162.0(11) |
| S2 | C11 | N3 | C12 | -2.4(11) | N62 | C70 | C68 | N61 | 6.5(12) |
| S2 | C11 | N2 | C10 | -16.1(13) | N62 | C70 | C68 | C67 | -174.7(11) |
| S61 | N61 | C68 | C70 | -139.6(8) | N42 | C50 | C48 | N41 | 1.8(10) |
| S61 | N61 | C68 | C67 | 41.7(10) | N42 | C50 | C48 | C47 | -178.8(9) |
| S61 | C61 | C66 | C67 | -6.8(9) | N22 | C30 | C28 | N21 | 8.5(13) |
| S61 | C61 | C66 | C65 | 174.6(8) | N22 | C30 | C28 | C27 | -177.6(10) |
| S61 | C61 | C62 | C63 | -174.5(9) | N2 | C10 | C8 | N1 | 5.5(11) |
| S41 | N41 | C48 | C50 | -140.0(7) | N2 | C10 | C8 | C7 | 179.4(9) |
| S41 | N41 | C48 | C47 | 40.6(8) | N61 | C68 | C67 | C66 | -2.8(14) |
| S41 | C41 | C46 | C47 | -7.4(10) | N41 | C48 | C47 | C46 | 0.3(11) |
| S41 | C41 | C46 | C45 | 171.6(7) | N21 | C28 | C27 | C26 | -2.6(12) |
| S41 | C41 | C42 | C43 | -173.9(9) | N1 | C8 | C7 | C6 | -7.7(11) |
| S21 | N21 | C28 | C30 | -145.8(7) | C70 | C68 | C67 | C66 | 178.5(10) |
| S21 | N21 | C28 | C27 | 39.9(9) | C50 | C48 | C47 | C46 | -179.2(9) |
| S21 | C21 | C26 | C27 | -8.3(10) | C30 | C28 | C27 | C26 | -176.5(10) |
| S21 | C21 | C26 | C25 | 177.3(9) | C10 | C8 | C7 | C6 | 178.5(10) |
| S21 | C21 | C22 | C23 | -176.7(10) | C68 | C67 | C66 | C65 | 163.0(12) |
| S1 | N1 | C8 | C10 | -139.3(7) | C68 | C67 | C66 | C61 | -15.6(13) |
| S1 | N1 | C8 | C7 | 46.5(8) | C48 | C47 | C46 | C45 | 164.0(9) |
| S1 | C1 | C6 | C7 | -5.3(8) | C48 | C47 | C46 | C41 | -17.0(11) |

| A | B | C | D | Angle/° | A | B | C | D | Angle/° |
|---|---|---|---|---|---|---|---|---|---|
| S1 | C1 | C6 | C5 | 171.7(7) | C28 | C27 | C26 | C25 | 160.7(11) |
| S1 | C1 | C2 | C3 | -173.2(8) | C28 | C27 | C26 | C21 | -13.2(14) |
| O63 | C70 | N62 | C71 | 5.2(14) | C8 | C7 | C6 | C5 | 168.8(10) |
| O63 | C70 | C68 | N61 | -176.1(11) | C8 | C7 | C6 | C1 | -14.2(11) |
| O63 | C70 | C68 | C67 | 2.6(14) | C67 | C66 | C65 | C64 | -179.4(10) |
| O43 | C50 | N42 | C51 | -2.5(14) | C67 | C66 | C61 | C62 | 175.5(9) |
| O43 | C50 | C48 | N41 | -172.5(10) | C47 | C46 | C45 | C44 | -175.2(10) |
| O43 | C50 | C48 | C47 | 6.9(12) | C47 | C46 | C41 | C42 | 173.5(10) |
| O23 | C30 | N22 | C31 | 2.6(15) | C27 | C26 | C25 | C24 | -175.3(12) |
| O23 | C30 | C28 | N21 | -173.2(11) | C27 | C26 | C21 | C22 | 170.0(10) |
| O23 | C30 | C28 | C27 | 0.7(15) | C7 | C6 | C5 | C4 | -177.2(9) |
| O3 | C10 | N2 | C11 | -4.3(12) | C7 | C6 | C1 | C2 | 176.4(8) |
| O3 | C10 | C8 | N1 | -172.7(9) | C66 | C65 | C64 | C63 | 5.2(14) |
| O3 | C10 | C8 | C7 | 1.2(10) | C66 | C61 | C62 | C63 | 3.1(12) |
| O67 | C67 | C68 | N61 | 175.9(12) | C46 | C45 | C44 | C43 | -1.8(12) |
| O67 | C67 | C68 | C70 | -2.8(14) | C46 | C41 | C42 | C43 | 5.1(13) |
| O67 | C67 | C66 | C65 | -15.9(13) | C26 | C25 | C24 | C23 | 6.6(14) |
| O67 | C67 | C66 | C61 | 165.6(10) | C26 | C21 | C22 | C23 | 5.1(12) |
| O47 | C47 | C48 | N41 | 178.7(10) | C6 | C5 | C4 | C3 | -3.4(11) |
| O47 | C47 | C48 | C50 | -0.8(12) | C6 | C1 | C2 | C3 | 5.1(10) |
| O47 | C47 | C46 | C45 | -14.5(11) | C65 | C64 | C63 | C62 | -5.4(13) |
| O47 | C47 | C46 | C41 | 164.5(9) | C45 | C44 | C43 | C42 | -0.7(14) |
| O27 | C27 | C28 | N21 | 178.6(10) | C25 | C24 | C23 | C22 | -5.9(14) |
| O27 | C27 | C28 | C30 | 4.7(14) | C5 | C4 | C3 | C2 | 1.9(11) |
| O27 | C27 | C26 | C25 | -20.4(13) | C64 | C63 | C62 | C61 | 1.3(14) |
| O27 | C27 | C26 | C21 | 165.7(9) | C44 | C43 | C42 | C41 | -0.9(14) |
| O7 | C7 | C8 | N1 | 176.1(9) | C24 | C23 | C22 | C21 | 0.0(14) |
| O7 | C7 | C8 | C10 | 2.3(11) | C4 | C3 | C2 | C1 | -2.8(11) |

**Table S18.** Hydrogen Atom Coordinates (Å×$10^4$) and Isotropic Displacement Parameters (Å$^2$×$10^3$) for **MLX-V.**

| Atom | *x* | *y* | *z* | U(eq) |
|---|---|---|---|---|
| H67 | 4590(30) | 4100(20) | 11326(15) | 27(11) |
| H47 | 3740(40) | 5480(11) | 3801(13) | 54(4) |
| H27 | 1410(40) | 6176(9) | 4554(6) | 50(3) |
| H7 | 440(30) | -1320(20) | 5970(30) | 59(4) |
| H62 | 3923(7) | 3870(7) | 8806(5) | 45(3) |

| Atom | *x* | *y* | *z* | U(eq) |
|---|---|---|---|---|
| H42 | 3679(7) | 4914(7) | 6765(5) | 44(4) |
| H22 | 1365(7) | 3243(7) | 5091(5) | 40(3) |
| H2 | 1000(7) | 1232(8) | 6028(6) | 54(4) |
| H74a | 73(10) | 5899(8) | 8790(20) | 63(4) |
| H74b | 665(9) | 6790(20) | 8690(30) | 63(4) |
| H74c | 356(18) | 6730(30) | 7745(7) | 63(4) |
| H54a | 2989(12) | 684(10) | 7880(30) | 74(5) |
| H54b | 4110(20) | 629(11) | 7550(20) | 74(5) |
| H54c | 3810(30) | 369(8) | 8656(11) | 74(5) |
| H34a | 1730(30) | 2340(30) | 9305(8) | 87(5) |
| H34b | 613(12) | 2100(40) | 9402(10) | 87(5) |
| H34c | 1440(40) | 1278(11) | 9524(8) | 87(5) |
| H14a | 4670(20) | 2192(11) | 3250(30) | 80(5) |
| H14b | 4917(14) | 1130(30) | 4068(8) | 80(5) |
| H14c | 4278(11) | 1270(30) | 3190(20) | 80(5) |
| H72 | 1611(7) | 5966(7) | 7033(6) | 36(3) |
| H52 | 3927(7) | 1865(7) | 9029(7) | 32(3) |
| H32 | 1103(8) | 983(8) | 8175(6) | 40(3) |
| H12 | 3368(8) | 2991(8) | 4025(6) | 43(3) |
| H65 | 6525(8) | 2999(8) | 12404(7) | 49(4) |
| H45 | 3896(7) | 7649(7) | 2231(6) | 31(3) |
| H25 | 1021(8) | 7765(7) | 2359(7) | 43(3) |
| H5 | -1458(7) | -2428(7) | 7042(6) | 36(3) |
| H64 | 7834(8) | 1863(7) | 12963(6) | 42(3) |
| H44 | 4053(9) | 9270(9) | 1471(8) | 49(4) |
| H24 | 1009(8) | 8530(8) | 718(7) | 42(3) |
| H4 | -2856(8) | -2919(8) | 8043(6) | 39(3) |
| H63 | 8329(8) | 985(9) | 12186(7) | 50(4) |

| Atom | *x* | *y* | *z* | U(eq) |
|---|---|---|---|---|
| H43 | 3838(9) | 10144(9) | 2358(7) | 52(4) |
| H23 | 1114(9) | 7632(8) | -151(7) | 50(4) |
| H3 | -3293(9) | -2222(9) | 9084(7) | 49(4) |
| H62a | 7412(7) | 983(7) | 10982(6) | 39(3) |
| H42a | 3466(7) | 9376(7) | 4013(6) | 33(3) |
| H22a | 1503(8) | 6014(8) | 615(6) | 48(4) |
| H2a | -2437(8) | -982(8) | 9032(6) | 38(3) |
| H69a | 6544(15) | 3760(20) | 9172(6) | 54(4) |
| H69b | 6210(20) | 3365(15) | 8451(16) | 54(4) |
| H69c | 5663(11) | 4300(12) | 8482(18) | 54(4) |
| H49a | 5055(10) | 6910(19) | 5338(9) | 53(4) |
| H49b | 4618(8) | 6690(20) | 6397(18) | 53(4) |
| H49c | 4965(11) | 5817(9) | 6140(20) | 53(4) |
| H29a | -111(11) | 4638(8) | 3110(20) | 58(4) |
| H29b | 365(9) | 3619(18) | 3220(20) | 58(4) |
| H29c | 18(13) | 3760(20) | 4161(7) | 58(4) |
| H9a | -1513(15) | 875(7) | 6210(20) | 49(4) |
| H9b | -1150(20) | 1577(15) | 6610(14) | 49(4) |
| H9c | -596(12) | 1515(18) | 5694(12) | 49(4) |

[1] L. Coppi, M. B. Sanmarti and M. C. Clavo, Crystalline forms of meloxicam and processes for their preparation and interconversion, patent US 2003/0109701 A1
